# Genomic Phylogenetic Analysis of *Physaliastrum* and *Archiphysalis* (Solanaceae): Insights From Chloroplast Genomes Indicate Distinct Evolutionary Relationships

**DOI:** 10.1002/ece3.71762

**Published:** 2025-07-07

**Authors:** Jiaju Xu, Zehuan Wang, Qianqian Zhong, Li Yan, Sirong Yi, Qingwen Sun

**Affiliations:** ^1^ Department of Traditional Chinese Medicine Resources and Development, College of Pharmacy Guizhou University of Traditional Chinese Medicine Guiyang Guizhou China; ^2^ Chongqing Three Gorges Medical College Chongqing Engineering Research Center of Antitumor Natural Drugs Chongqing China

**Keywords:** *Archiphysalis*, chloroplast genome, phylogenetic relationship, *Physaliastrum*, Withaninae

## Abstract

This research involved the sequencing and analysis of chloroplast genomes from nine species across four genera of the Solanaceae family, specifically two species of *Archiphysalis*, five species of *Physaliastrum*, and the taxa *Tuberowithania pengiana* and *Tubocapsicum anomalum*. We conducted a comprehensive series of analyses, including assessments of genome structure, gene content, codon usage, selection pressure, and other genomic features. The chloroplast genomes of *Archiphysalis* and *Physaliastrum* exhibit typical quadripartite structures, with lengths ranging from 156,345 to 156,924 bp. Notably, *Archiphysalis* contains 130 genes, while the species of *Physaliastrum* have a slightly higher gene count of 132. Phylogenetic analysis reveals that *A. sinensis* and *A. chamaesarachoides* cluster together within the subtribe Withaninae, which is consistent with their fruit morphology. Divergence time analysis indicates that *Physaliastrum* separated from *Archiphysalis* approximately 41.2 million years ago (95% HPD: 38.6–43.5 Mya), resulting in intergeneric differences in chloroplast gene features that are significantly greater than intrageneric differences, particularly in gene count, structure, and lengths of gene duplications. These findings provide genomic‐level support for the classification of species within these genera and underscore the close relationship between *A. chamaesarachoides* and *A. sinensis* .

## Graphical abstract

This research involved the sequencing and analysis of chloroplast genomes from nine species across four genera of the Solanaceae family, specifically two species of Archiphysalis, five species of *Physaliastrum*, and the taxa *Tuberowithania pengiana* and *Tubocapsicum anomalum*. We conducted a phylogenetic analysis that clarifies the relationships among these genera and confirms their distinct generic statuses. Additionally, this study provides genomic evidence supporting their classification and highlights the close relationship between *A. sinensis* and *A. chamaesarachoides*.

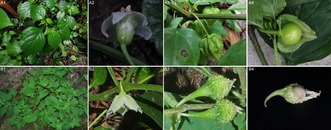

## 1 Introduction

The genus *Physaliastrum* Makino was first established by Makino in 1914, initially comprising two Japanese species: *P. echinatum* (Franch. et Sav.) Honda and *P. savatieri* Makino. These species were originally classified under the American genus *Chamaesaracha* (A.Gray) Benth. & Hook.f., but were later transferred to *Physaliastrum* due to distinct morphological characteristics, including a campanulate (not rotate) corolla, induplicato‐vatvate (not plicate) corolla lobes, and a much enlarged fleshy persistent calyx (Makino 1914). Over time, the genus *Physaliastrum* expanded to include a total of nine species. Among these, five are exclusively endemic to China: *P. heterophyllum* (Hemsl.) Migo, *P. kweichouense* Kuang & A.M.Lu, *P. sinense* (Hemsl.) D'Arcy & Z.Y.Zhang (syn. *Archiphysalis sinensis* (Hemsl.) Kuang), *P. sinicum* Kuang & A.M.Lu, and *P. yunnanense* Kuang & A.M.Lu. Additionally, *P. chamaesarachoides* (syn. *A. chamaesarachoides* (Makino) Kuang) and *P. echinatum* are widely distributed across China and Japan, with the latter also found in Korea and Russia. The remaining two species, *P. kimurai* Makino and *P. savatieri* , are found only in Japan (Kuang and Lu 1965; Zhang et al. 1994).

**FIGURE 1:**
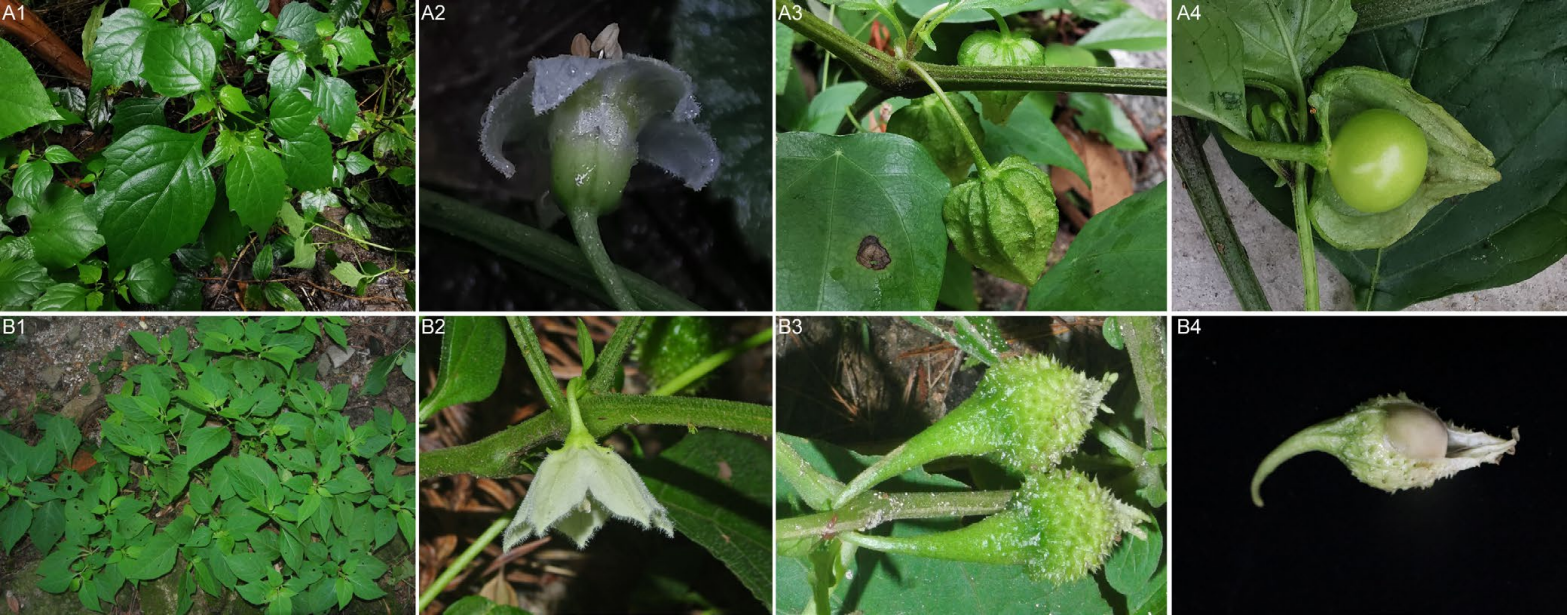
Morphological images of *Archiphysalis sinensis* (top: A1–A4) and *Physaliastrum heterophyllum* (bottom: B1–B4).

In 1966, Kuang established the genus *Archiphysalis* Kuang, initially including three species: *A. chamaesarachoides*, *A. kwangsiensis* Kuang, and *A. sinensis* , and distinguished it from *Physaliastrum* based on the degree to which the berry fills the fruiting calyx (Kuang 1966; Kuang and Lu 1978). Species of *Archiphysalis* were characterized by a bladdery calyx that significantly exceeds the berry in size, whereas *Physaliastrum* typically features a spiny calyx that closely adheres to the fruit (Figure 1). However, this distinction was later challenged. D'Arcy and Zhang (1992) argued that the variation in calyx morphology was insufficient to justify the separation of *Archiphysalis* from *Physaliastrum*. Consequently, Zhang et al. (1994) reclassified all species of *Archiphysalis* back into *Physaliastrum*, merging *A. kwangsiensis* and *A. chamaesarachoides* into a single species, *P. chamaesarachoides* (Makino) Makino.

However, the classification of *Archiphysalis sinensis* or *A. chamaesarachoides* within *Physaliastrum* has not been supported by subsequent phylogenetic analyses. Li et al. (2013) used three DNA sequences (ITS, *ndhF*, and *trnL‐F*) to analyze the taxonomic positions of *P. heterophyllum* and *A. chamaesarachoides*. Their results revealed that these two species belong to different subtribes within Solanaceae: *A. chamaesarachoides* is placed in the subtribe Withaninae Bohs & Olmstead, while *P. heterophyllum* is classified within the subtribe Physalinae Reveal. This supports the independent status of *Archiphysalis* from *Physaliastrum*, which has also been corroborated by Huang's recent research based on orthologous genomic data (Huang et al. 2023).

Research by Deanna et al. (2019) utilized four DNA markers (ITS, *LEAFY*, *trnL‐F*, and *waxy*) to conduct a phylogenetic study of the tribe Physalideae. Their results supported the independent status of *A. chamaesarachoides* as the sole member of the genus *Archiphysalis*, whereas the type species *A. sinensis* remained classified under *Physaliastrum*. In contrast, a later study by Wang et al. (2024), based on the same datasets but including the newly described genus *Tuberowithania* Ze H.Wang & Yi Yang, did not support this finding. This latter study revealed that, although the two species formerly placed in *Archiphysalis* did not cluster together, they each formed distinct branches with other genera of the subtribe Withaninae.

In summary, current molecular systematic studies rely on a limited number of genetic markers (Li et al. 2013) and primarily focus on broader taxonomic levels, such as the tribe Physalideae (Deanna et al. 2019; Wang et al. 2024) or the family Solanaceae (Huang et al. 2023), resulting in limited representation of samples from the genera *Physaliastrum* and *Archiphysalis*. Additionally, the morphological similarities between *A. chamaesarachoides* and *A. sinensis* (Kuang 1966) raise questions about their classification within different subtribes or lineages of the subtribe Withaninae. Furthermore, the studies by Deanna et al. (2019) and Wang et al. (2024) indicate inconsistencies in the phylogenetic relationships revealed by different genetic markers, highlighting the ongoing complexity and unresolved nature of their taxonomic relationships. This highlights the need for further research to determine their definitive systematic positions.

In this study, we utilize next‐generation sequencing technology to obtain complete chloroplast genome sequences for *Archiphysalis sinensis*, *A. chamaesarachoides*, and five species of *Physaliastrum*. Our goal is to elucidate the systematic relationships among these seven species, with a particular focus on the two species of *Archiphysalis*.

## 2 Materials and Methods

### 2.1 Plant Material Information, Sequencing, Assembly, and Annotation

To investigate the relationships between *Archiphysalis* and *Physaliastrum*, we conducted field surveys for these genera and their related taxa *Tuberowithania* and *Tubocapsicum* (Wettst.) Makino. Mature leaves were collected from individuals at these localities and dried using silica gel, with voucher specimens deposited in the herbarium of Guizhou University of Traditional Chinese Medicine, China (GZTM). All samples were sent to the Molecular Biology Experiment Center, Germplasm Bank of Wild Species, Kunming, China, for DNA extraction and library preparation. The libraries were sequenced on the DNBSEQ‐T7 platform, yielding 150 bp paired‐end reads and approximately 20 G of raw data.

The sequencing results underwent quality control using fastp v0.23.2 (Chen et al. 2018) with default settings, which involved removing adapters and filtering out low‐quality sequences to obtain high‐quality next‐generation sequencing (NGS) data. The NGS data were then assembled into chloroplast genomes with GetOrganelle v1.7.5 (Jin et al. 2020), using the genomes with the highest BLAST values as references: *Withania* sp. (OR426647) for *Physaliastrum*, and *Nothocestrum latifolium* (OR400642) for *Archiphysalis*, *Tuberowithania pengiana* Ze H.Wang & Yi Yang, and *Tubocapsicum anomalum* (Franch. & Sav.) Makino.

Annotation of the chloroplast genomes was performed using GeSeq (Tillich et al. 2017) and further manually corrected with Geneious Prime 9.0.2 (Kearse et al. 2012), incorporating insights from Qu et al. (2023). The physical map of the chloroplast genome was constructed using OrganellarGenomeDRAW (https://chlorobox.mpimp‐golm.mpg.de/OGDraw.html, Lohse et al. 2007), and the annotated protein‐coding sequences, rRNA genes, and tRNA genes were summarized using CPJSdraw (Li et al. 2023). Finally, the chloroplast genome sequences were deposited in NCBI with accession numbers PV472654–PV472662.

### 2.2 Phylogenetic Analysis

To enhance the reliability of our results, we included *Tuberowithania pengiana* and *Tubocapsicum anomalum* in our phylogenetic analysis, along with two species of *Archiphysalis* and five species of *Physaliastrum*, as both *Tuberowithania* and *Tubocapsicum* are closely related to *Archiphysalis* (Deanna et al. 2019; Wang et al. 2024). We retrieved 47 chloroplast genomes from the NCBI database (https://www.ncbi.nlm.nih.gov/) based on Deanna et al. (2019) and excluded four sequences: *T. anomalum* (MW829600) and *Withania somnifera* (MN746302, MK142783, OR166175). MK142783 and OR166175 were excluded for aligning with our selected OR166174, simplifying the dataset. MW829600 and MN746302 were removed due to concerns about their accuracy, as the phylogenetic trees (Figures T1 and T2: Appendix S1) showed inconsistent placements within the Physalideae compared to the results of several published studies (Olmstead et al. 2008; Li et al. 2013; Deanna et al. 2019; Huang et al. 2023; Wang et al. 2024). We resampled *T. anomalum* to improve data reliability. This resulted in a total of 56 genomes, including 47 from NCBI and 9 newly sequenced species, which were aligned using MAFFT in PhyloSuite v1.2.3 (Zhang et al. 2020). *Capsicum lycianthoides* (NC_026551) and *Lycianthes radiata* (NC_062492) served as outgroups, following the methodology and findings of Deanna et al. (2019).

Phylogenetic trees were constructed using both Bayesian Inference (BI) and Maximum Likelihood (ML) methods. The best substitution model for both analyses was determined using ModelFinder in PhyloSuite v1.2.3 (Zhang et al. 2020). For the Bayesian method, the best‐fit model selected according to the Bayesian Information Criterion (BIC) was GTR + F + I + G4. For the maximum likelihood method, the best‐fit model chosen based on the Akaike Information Criterion (AIC) was TVM + F + I + I + R6. Subsequently, phylogenetic trees were built using MrBayes and IQ‐TREE within the same software, applying the selected model, with MrBayes running for 2,000,000 generations and IQ‐TREE for 1000 generations, while all other parameters were set to their defaults. Finally, we visualized and edited the phylogenetic trees using FigTree v.1.4.4 (http://tree.bio.ed.ac.uk/software/figtree/, Rambaut 2018).

### 2.3 Divergence Time Estimation

The MCMCtree module in PAML v4.9 (Yang 2007) was employed to estimate divergence times among species based on the phylogenetic tree constructed from 56 chloroplast genomes. To calibrate this tree, we incorporated three estimated divergence times: the tribe Capsiceae (31.0–45.4 Mya), the tribe Physalideae (27.8–42.7 Mya), and the combined divergence time for both tribes (52.2 Mya), as reported by Deanna et al. (2019). Additionally, we referenced a fossil of a lantern fruit, which exhibits features characteristic of Physalideae within Solanaceae, dating back to the early Eocene (ca. 52 Ma) from the Laguna del Hunco site in Chubut, Argentina (Deanna et al. 2020; Wilf et al. 2017). Following these estimations, we used Tracer v. 1.6 (Rambaut et al. 2018) to assess parameter convergence, ensuring that Effective Sample Size (ESS) values exceeded 200.

### 2.4 Genomic Structure Comparison and Sequence Divergence Analysis

To explore the structural variations in the chloroplast genomes of *Archiphysalis* and *Physaliastrum*, we analyzed the genome size, lengths of the LSC, SSC, and IR regions, GC content, and gene numbers of the seven species using Geneious v9.0.2 (Kearse et al. 2012), along with the scripts calculate_gc.py and count_genes.py from CPStools 2.0.9 (Huang et al. 2024). We utilized the online tool IRscope (https://irscope.shinyapps.io/irapp/, Amiryousefi et al. 2018) to investigate the expansion and contraction of the Inverted Repeat (IR) regions. To assess sequence divergence between the two species of *Archiphysalis* and five species of *Physaliastrum*, we employed mVISTA (https://genome.lbl.gov/vista/index.shtml, Frazer et al. 2004) in Shuffle–LAGAN mode, using the chloroplast genome of *P. kweichouense*, obtained in this study as the reference.

Following this analysis, we aligned the chloroplast genome sequences of the seven species using MAFFT within Geneious Prime 9.0.2 (Kearse et al. 2012). To further investigate sequence divergence and identify mutation hotspots, we conducted a nucleotide diversity analysis with DnaSP v6 (Rozas et al. 2017), setting the sliding window to a length of 600 bp and a step size of 200 bp.

### 2.5 Repeat Analysis

Simple sequence repeats (SSRs) were identified using the SSRS_annotation.py script in CPStools 2.0.9 (Huang et al. 2024), which effectively categorizes the types of c and c* repeat sequences. To further analyze repeat sequences in the chloroplast genome, we employed the online tool REPuter (https://bibiserv.cebitec.uni‐bielefeld.de/reputer, Kurtz et al. 2001). This tool facilitated the identification of forward, reverse, complementary, and palindromic repeat sequences, with parameters set to a maximum repeat length of 5000 bp, a Hamming distance of 3, and a minimum repeat length of 30 bp.

### 2.6 Condon Usage Analysis

To investigate codon usage preferences and influencing factors in the chloroplast genomes of *Archiphysalis* and *Physaliastrum*, we utilized the rscu.py script from CPStools 2.0.9 (Huang et al. 2024) to analyze codon counts and relative synonymous codon usage (RSCU) values. The script excludes sequences shorter than 300 bp and ensures that all analyzed sequences use ATG as the start codon and either TAA, TAG, or TGA as stop codons. An RSCU value of 1 indicates no codon preference, while values greater than 1 indicate a strong preference, and values less than 1 indicate lesser usage.

To analyze codon usage bias and its driving factors, we generated ENC‐GC_3s_, Neutrality, and PR2‐bias plots. ENC‐GC_3s_ plots reveal whether codon usage bias is driven by mutation (genes along the standard curve) or selection (genes below the curve). The expected ENC baseline is established through a theoretical curve, and points clustering near this curve suggest mutation is the primary driver, whereas deviations indicate that natural selection may play a significant role (Novembre 2002). Additional analyses using discrepancies between empirical and predicted ENC values further quantify these influences (Kawabe and Miyashita 2003; Zhang et al. 2007).

Neutrality plots assess the correlation between GC content at different codon positions to distinguish between mutation and selection effects. PR2‐bias plots examine preferences for A/T and C/G, with deviations from the center point indicating the degree and direction of codon usage bias. Together, these analyses elucidate the factors affecting codon usage in chloroplasts.

### 2.7 Selection Pressure Analysis

To reveal the types of selection pressures and their differences experienced by the genes of *Archiphysalis*, *Tuberowithania*, *Tubocapsicum*, and *Physaliastrum* during evolution, we conducted an intergeneric selection pressure analysis. The nonsynonymous to synonymous substitution rate (Ka/Ks) values were calculated using the Genepioneer platform (http://112.86.217.82:9929/#/tool/alltool/detail/305) provided by Nanjing Jisi Huiyuan Biotechnology Co. Ltd. We used the annotated gb file of *P. kweichouense* as the reference and imported the annotated gb files of *Archiphysalis*, *Tuberowithania*, and *Tubocapsicum* as input files for comparative analysis. By utilizing the platform's built‐in tools, including BLASTN v2.10.1, MAFFT v7.427 (Katoh and Standley 2013), and Perl scripts, we automated the calculation of Ka/Ks values between the input and reference files, selecting genetic code table 11 (bacterial and plant plastid code) and employing the Maximum Likelihood with Weighted Likelihood (MLWL) method.

## 3 Results

### 3.1 Features of the Chloroplast Genome

**FIGURE 2:**
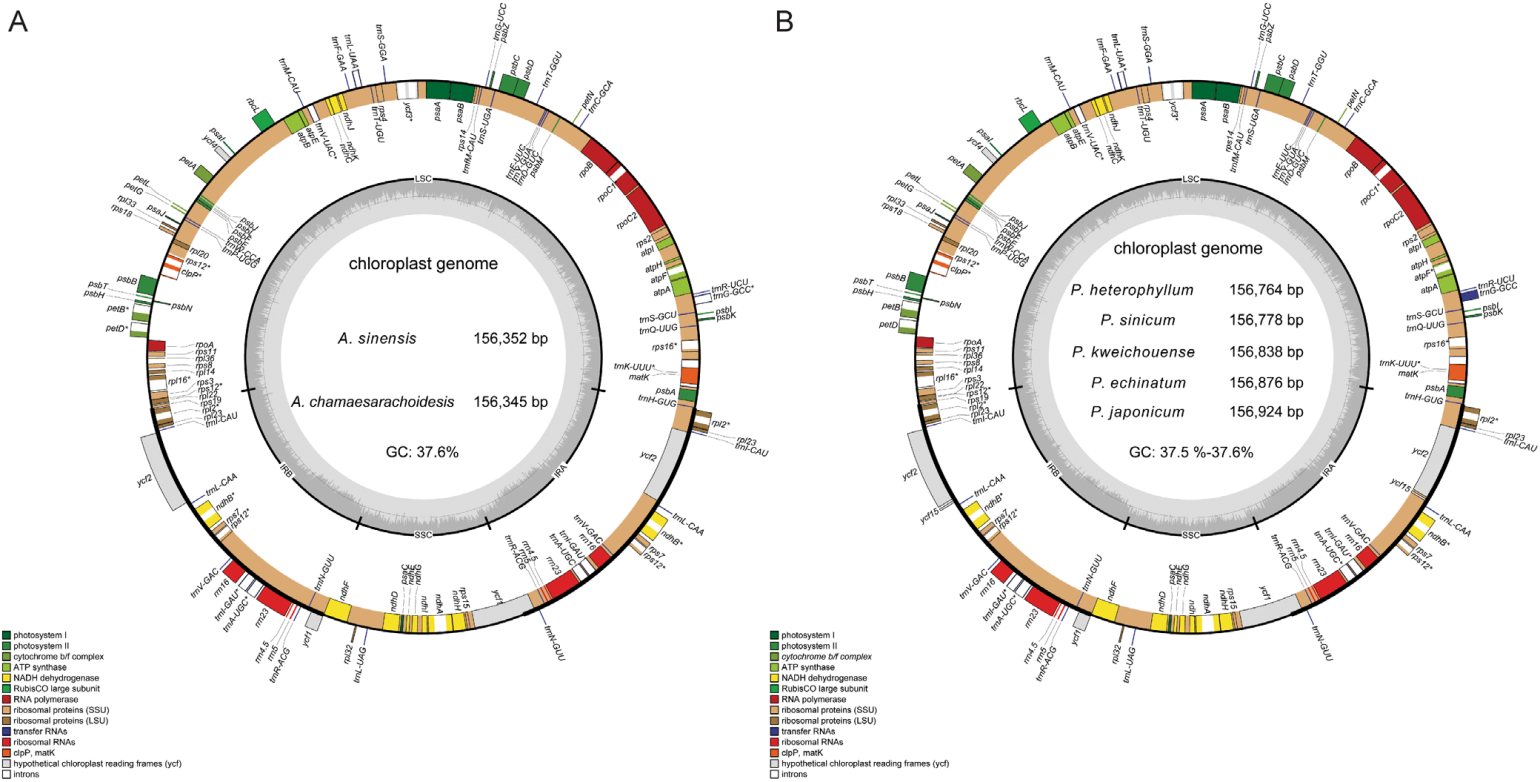
Gene map of the chloroplast genomes of *Archiphysalis* (A) and *Physaliastrum* (B). Genes inside the outer circle are transcribed clockwise, while those outsides are transcribed counterclockwise. Genes are color‐coded according to different functional groups. The darker gray in the inner circle indicates the GC content, and the lighter gray indicates the AT content. The inner circle also shows that the chloroplast genome contains two copies of inverted repeats (IRA and IRB), a large single‐copy (LSC) region, and a small single‐copy (SSC) region.

The complete chloroplast genomes of two *Archiphysalis* species and five *Physaliastrum* species have been successfully assembled and annotated. All seven genomes exhibit a circular DNA molecule characterized by a typical quadripartite structure, as illustrated in Figure 2. This structure comprises a large single‐copy (LSC) region from 86,944 to 87,450 bp, a small single‐copy (SSC) region from 18,435 to 18,565 bp, and a pair of inverted repeat (IR) regions spanning from 50,446 to 51,308 bp. The total length of these genomes varies from 156,345 to 156,924 bp, with an overall GC content of 37.5%–37.6%. GC content is unevenly distributed among the four genomic regions: the LSC region exhibits a GC content of 35.58% to 35.67%, while the SSC region has the lowest content, ranging from 31.40% to 31.89%. In contrast, the IR regions have the highest content, between 43.05% and 43.12%. Additionally, the GC content in the LSC and SSC regions of the two *Archiphysalis* species is higher than that in *Physaliastrum*, while the IR region in these two species exhibits a lower GC content compared to *Physaliastrum* (Table 1).

**TABLE 1:** The length and composition of the complete chloroplast genome of *Archiphysalis* and *Physaliastrum*.

Species	Length (bp)				GC%				PCG	tRNA	rRNA	Total genes
	cp. genome	LSC	SSC	IR	cp. genome	LSC	SSC	IR				
*A. sinensis*	156,352	87,442	18,444	50,446	37.60	35.66	31.90	43.05	85	37	8	130
*A. chamaesarachoides*	156,345	87,450	18,435	50,460	37.60	35.67	31.89	43.05	85	37	8	130
*P. heterophyllum*	156,764	86,944	18,514	51,306	37.60	35.58	31.45	43.10	87	37	8	132
*P. kweichouense*	156,838	86,993	18,539	51,306	37.60	35.58	31.44	43.10	87	37	8	132
*P. sinicum*	156,778	86,948	18,522	51,308	37.60	35.59	31.41	43.10	87	37	8	132
*P. echinatum*	156,876	87,038	18,548	51,290	37.60	35.59	31.43	43.12	87	37	8	132
*P. japonicum*	156,924	87,069	18,565	51,290	37.50	35.58	31.40	43.11	87	37	8	132

Annotation of the chloroplast genomes revealed that the two *Archiphysalis* species contain 130 genes, including 85 protein‐coding genes (PCGs), 37 tRNA genes, and 8 rRNA genes. In contrast, the five *Physaliastrum* species have 132 genes, which possess two copies of the *ycf15* gene compared to those of the *Archiphysalis* species (Table 2). Based on their functions, these genes are categorized into four major groups: genes related to photosynthesis (44 genes), self‐replication (59 genes), other functional genes (including *matK*, *clpP*, *cemA*, *accD*, *ccsA*), and genes of unknown function (*ycf1*, *ycf2*, *ycf3*, *ycf4*, *ycf15*). Among all the annotated genes, 15 contain one intron, 3 genes (*rps12*, *clpP*, *ycf3*) contain two introns, and 18 genes are duplicated due to their presence in the IR regions. Specifically, the genes *ndhB*, *rpl2*, *trnA‐UGC*, and *trnI‐GAU* contain one intron and are duplicated, while the gene *rps12*, which contains two introns, is also duplicated, with its 5′ and 3′ exons located in the LSC and IR regions, respectively.

**TABLE 2:** List of genes identified in the seven complete chloroplast genomes of *Archiphysalis* and *Physaliastrum*.

Category of genes	Group of genes	Name of genes
Photosynthesis	Subunits of photosystem I	*psaA*, *psaB*, *psaC*, *psaI*, *psaJ*
	Subunits of photosystem II	*psbA*, *psbB*, *psbC*, *psbD*, *psbE*, *psbF*, *psbH*, *psbI*, *psbJ*, *psbK*, *psbL*, *psbM*, *psbN*, *psbT*, *psbZ*
	Subunits of NADH dehydrogenase	*ndhA*,^a^ *ndhB* ^a^ (×2), *ndhC*, *ndhD*, *ndhE*, *ndhF*, *ndhG*, *ndhH*, *ndhI*, *ndhJ*, *ndhK*
	Subunits of cytochrome b/f complex	*petA*, *petB*,^a^ *petD*,^a^ *petG*, *petL*, *petN*
	Subunits of ATP synthase	*atpA*, *atpB*, *atpE*, *atpF*,^a^ *atpH*, *atpI*
	Large subunit of rubisco	*rbcL*
Self‐replication	Large ribosomal subunit	*rpl14*, *rpl16*,^a^ *rpl2* ^a^ (×2), *rpl20*, *rpl22*, *rpl23*(×2), *rpl32*, *rpl33*, *rpl36*
	Small ribosomal subunit	*rps11*, *rps12* ^b^ (×2), *rps14*, *rps15*, *rps16*,^a^ *rps18*, *rps19*, *rps2*, *rps3*, *rps4*, *rps7*(×2), *rps8*
	RNA polymerase	*rpoA*, *rpoB*, *rpoC1*,^a^ *rpoC2*
	Ribosomal RNAs	*rrn16*(×2), *rrn23*(×2), *rrn4.5*(×2), *rrn5*(×2)
	Transfer RNAs	*trnA‐UGC* ^a^ (×2), *trnC‐GCA*, *trnD‐GUC*, *trnE‐UUC*, *trnF‐GAA*, *trnG‐GCC*,^a^ *trnG‐UCC*, *trnH‐GUG*, *trnI‐CAU*(×2), *trnI‐GAU* ^a^ (×2), *trnK‐UUU*,^a^ *trnL‐CAA*(×2), *trnL‐UAA*,^a^ *trnL‐UAG*, *trnM‐CAU*, *trnN‐GUU*(×2), *trnP‐UGG*, *trnQ‐UUG*, *trnR‐ACG*(×2), *trnR‐UCU*, *trnS‐GCU*, *trnS‐GGA*, *trnS‐UGA*, *trnT‐GGU*, *trnT‐UGU*, *trnV‐GAC*(×2), *trnV‐UAC*,^a^ *trnW‐CCA*, *trnY‐GUA*, *trnfM‐CAU*
Other function	Maturase	*matK*
	Protease	*clpP* ^b^
	Envelope membrane protein	*cemA*
	Acetyl‐CoA carboxylase	*accD*
	c‐type cytochrome synthesis gene	*ccsA*
Unknown function	Conserved open reading frames	*ycf1, ψycf1*, *ycf2*(×2), *ycf3*,^b^ *ycf4*, ^1^ *ycf15*(×2)

*Note:* (×2): duplicated gene; 1: gene only present in *Physaliastrum*; *ψ*: pseudogene.

^a^ Gene contains one intron.

^b^ Gene contains two introns.

### 3.2 Phylogenetic Analysis

In this study, we successfully assembled the complete chloroplast genomes of both species of *Archiphysalis* and five out of seven species of *Physaliastrum*, facilitating an exploration of the phylogenetic relationships among these genera. Furthermore, we also sequenced and obtained the complete chloroplast genomes of *Tuberowithania pengiana* and *Tubocapsicum anomalum*, as previous studies have indicated their close relationship with *Archiphysalis* (Deanna et al. 2019; Wang et al. 2024). Sampling these two genera is essential for accurately determining the phylogenetic placement of the *Archiphysalis* species. Detailed collection information for all newly sequenced samples is provided in Table 3.

**TABLE 3:** Sampling information of the nine newly generated chloroplast genomes.

Species	Collector and collection no.	Location	GenBank accession no.
*Archiphysalis sinensis*	Yi Sirong YSR202311‐01	Nanchuan District, Chongqing, China	PV472654
*Archiphysalis chamaesarachoides*	Li Hongqing et al. Lihq0393	Kuaihua County, Quzhou City, Zhejiang Province, China	PV472655
*Physaliastrum heterophyllum*	Wang Zehuan 202307‐01	Qianshan City, Anqing City, Anhui Province, China	PV472656
*Physaliastrum kweichouense*	Sangzhi Forestry Science Institute 0995	Sangzhi County, Zhangjiajie City, Hunan Province, China	PV472657
*Physaliastrum sinicum*	Liu Bing, Feng Yalei, Zhou Xinxin 12357	Fangshan District, Beijing City, China	PV472658
*Physaliastrum echinatum*	Wang Zehuan 202408‐01	Huairou District, Beijing City, China	PV472659
*Physaliastrum japonicum*	Miyoshi Furuse 44638	Yama‐kita‐choo, Ashigara‐kami‐gun, Kanagawa Prefecture, Japan	PV472660
*Tubocapsicum anomalum*	Wang Zehuan & Xu Jiaju 202407‐01	Wulingyuan District, Zhangjiajie City, Hunan Province, China	PV472661
*Tuberowithania pengiana*	Wang Zehuan & Chen Li 202208‐01	Shuangjiang County, Lincang City, Yunnan Province, China	PV472662

To construct a robust dataset for phylogenetic analysis, we retrieved 47 chloroplast genomes from the NCBI database (https://www.ncbi.nlm.nih.gov/) based on the phylogenetic research conducted by Deanna et al. (2019) on the Physalideae tribe. This resulted in a final dataset comprising 56 genomes, including two outgroup species: *Capsicum lycianthoides* (NC_026551) and *Lycianthes radiata* (NC_062492). The final aligned dataset of chloroplast genomes for phylogenetic analysis spans 165,714 bp, including 3692 parsimony‐informative sites.

**FIGURE 3:**
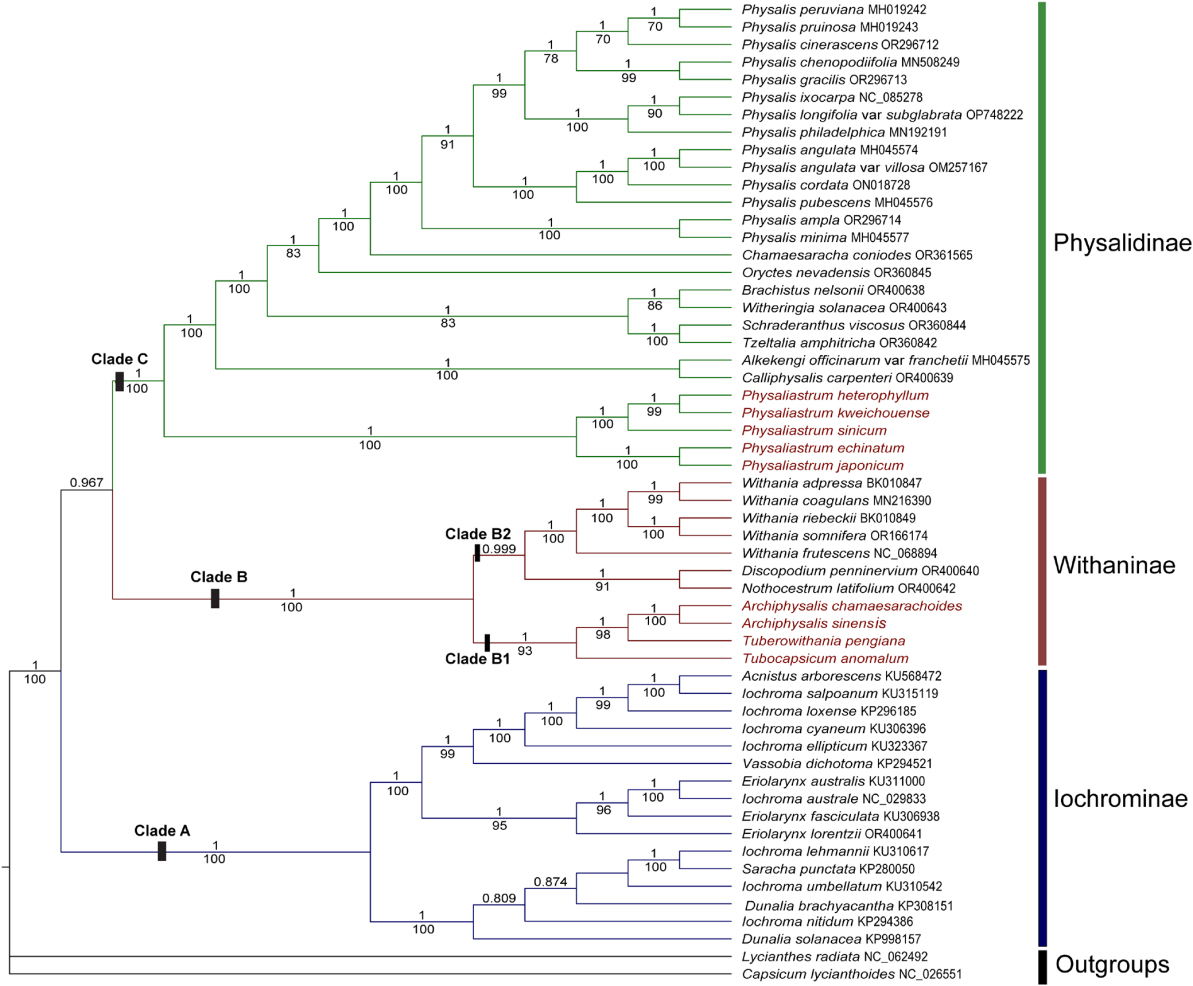
The BI tree topology of the phylogenetic cladogram, based on whole chloroplast genome sequences of 56 taxa, displays posterior probabilities (PP > 0.7) above the branches and Maximum Likelihood bootstrap values (BS > 70) below. Newly sequenced samples are labeled solely with species names, while other downloaded genomes include both species names and NCBI accession numbers.

The phylogenetic trees constructed using the Bayesian Inference (BI) and Maximum Likelihood (ML) methods exhibit identical topologies, though there are minor differences in branch support values. Through the analysis of chloroplast genomes, we have clearly resolved the phylogenetic relationships within the genera *Archiphysalis* and *Physaliastrum*, with particular emphasis on the phylogenetic position of *A. sinensis* . As illustrated in Figure 3, we identified three subtribes within the tribe Physalideae: Iochrominae Reveal, Withaninae, and Physalinae. Notably, the five species of *Physaliastrum* are nested within the Physalinae subtribe, while *A. chamaesarachoides* is placed in the Withaninae subtribe, consistent with previous studies (Deanna et al. 2019; Huang et al. 2023; Li et al. 2013; Wang et al. 2024).

However, our findings also indicate a significant discrepancy from previous studies (Deanna et al. 2019; Huang et al. 2023; Li et al. 2013; Wang et al. 2024), as *Archiphysalis sinensis*, the type species of the genus *Archiphysalis*, clusters with *A. chamaesarachoides* within the subtribe Withaninae (BS = 100, PP = 1), rather than grouping with *Physaliastrum*. This clustering, which aligns with our expectations, forms a single clade alongside *Tuberowithania pengiana* and *Tubocapsicum anomalum* (BS = 93, PP = 1).

### 3.3 Divergence Time Estimation

**FIGURE 4:**
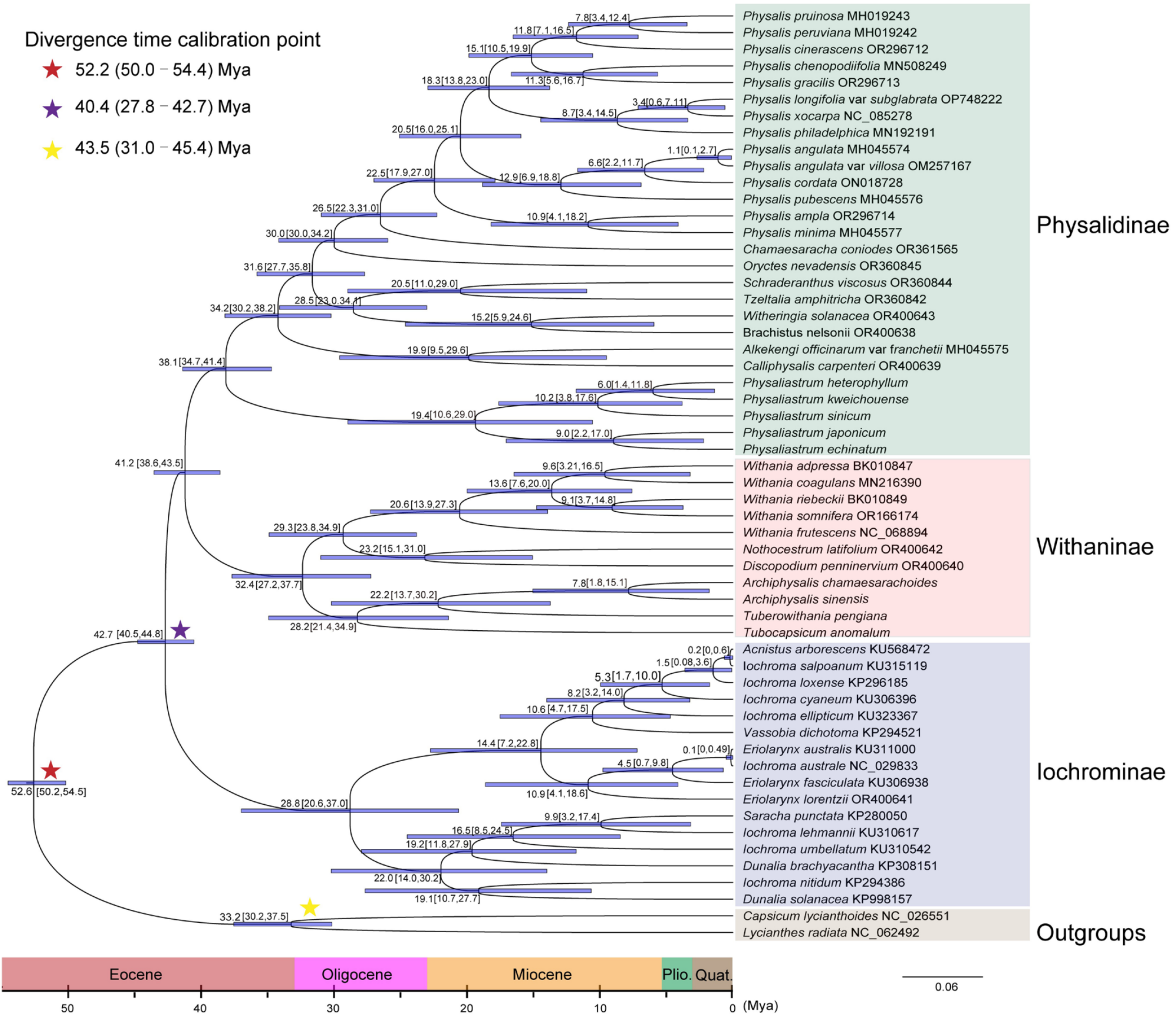
Divergence times of major clades within the Physalideae tribe. This chronogram was derived from the MCMCtree module in PAML, based on whole chloroplast genome sequences. Three calibration points are indicated by stars. The blue bars represent the 95% highest posterior density intervals for node ages, while the mean ages for each node are displayed above the respective bars.

The differentiation times of the major clades within the Physalideae tribe are illustrated in Figure 4. Divergence time estimates indicate that the crown age of the clade formed by the Physalinae and Withaninae subtribes separated during the middle Eocene, specifically around 41.2 million years ago (Mya) (95% HPD: 38.6–43.5 Mya). Within the Physalinae subtribe, five species of *Physaliastrum* constitute a basal clade, with a stem age dating back to the late Eocene at approximately 38.1 Mya (95% HPD: 34.7–41.4 Mya), and the crown age estimated to be around 19.4 Mya (95% HPD: 10.6–29.0 Mya) in the early Miocene.

In the Withaninae subtribe, the two *Archiphysalis* species, along with *Tuberowithania pengiana* and *Tubocapsicum anomalum*, form a sister clade to all other members of the Withaninae subtribe. This clade has a stem age from the late Eocene, estimated at 32.4 Mya (95% HPD: 27.2–37.7 Mya), and a crown age of approximately 28.2 Mya (95% HPD: 21.4–34.9 Mya) in the middle Oligocene. For the two *Archiphysalis* species, the stem age is estimated to be 22.2 Mya (95% HPD: 13.7–30.2 Mya), while their crown age is around 7.8 Mya (95% HPD: 1.8–15.1 Mya).

### 3.4 Comparative Genomic Analysis

#### 3.4.1 IR Contraction and Expansion

**FIGURE 5:**
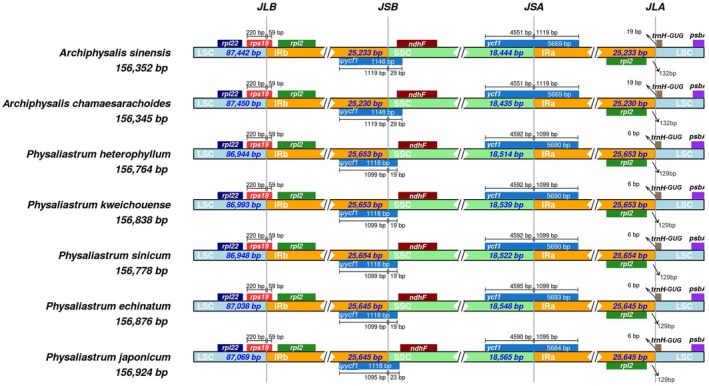
Comparison of LSC, SSC, and IR junction positions in the chloroplast genomes of *Archiphysalis* and *Physaliastrum*. The junctions are defined as follows: JLB (LSC/IRb), JSB (SSC/IRb), JSA (SSC/IRa), and JLA (LSC/IRa). Genes transcribed forward are shown above the lines, while those transcribed reverse are shown below. Gene lengths in each region are indicated above the corresponding gene boxes.

A comparative analysis of the inverted repeat (IR) boundaries was conducted on the complete chloroplast genomes of seven species from the genera *Physaliastrum* and *Archiphysalis*. The results, illustrated in Figure 5, indicate that the boundary genes at JLB (LSC/IRb), JSB (IRb/SSC), JSA (SSC/IRa), and JLA (IRa/LSC) exhibit complete identity across the species, demonstrating a high degree of conservation. Overall, the IR region of *Physaliastrum* is longer than that of *Archiphysalis*, measuring between 415 and 421 bp. Additionally, the IR lengths among different species within each genus are relatively consistent, differing by 8–9 and 3 bp, respectively.

Further observations have shown that the *rps19* gene, which spans the LSC/IRb boundary, consistently measures 220 bp in the LSC region and 59 bp in the IRb region across all species. Similarly, the IRa/LSC boundary is consistently located between the *rpl2* and the *trnH‐GUG* genes in the analyzed chloroplasts. In *Archiphysalis*, the distances between the IRa/LSC boundary and the *rpl2* and *trnH‐GUG* genes are 132 and 19 bp, respectively, whereas in *Physaliastrum*, these distances are 129 and 6 bp. In contrast, the *ycf1* gene, which spans the IRb/SSC and SSC/IRa boundaries, demonstrates variability in length among species. Specifically, in the five *Physaliastrum* species, the *ycf1* gene ranges from 5684 to 5693 bp in the IRb/SSC region, while the two *Archiphysalis* species consistently measure 5669 bp. Additionally, at the JSA boundary, the pseudogene *ψycf1* has lengths of 1118 bp in *Physaliastrum* and 1148 bp in *Archiphysalis*, both extending into the SSC region by 19–23 and 29 bp, respectively (Figure 5).

#### 3.4.2 Comparison of Chloroplast Genomes and Hotspot Identification

**FIGURE 6:**
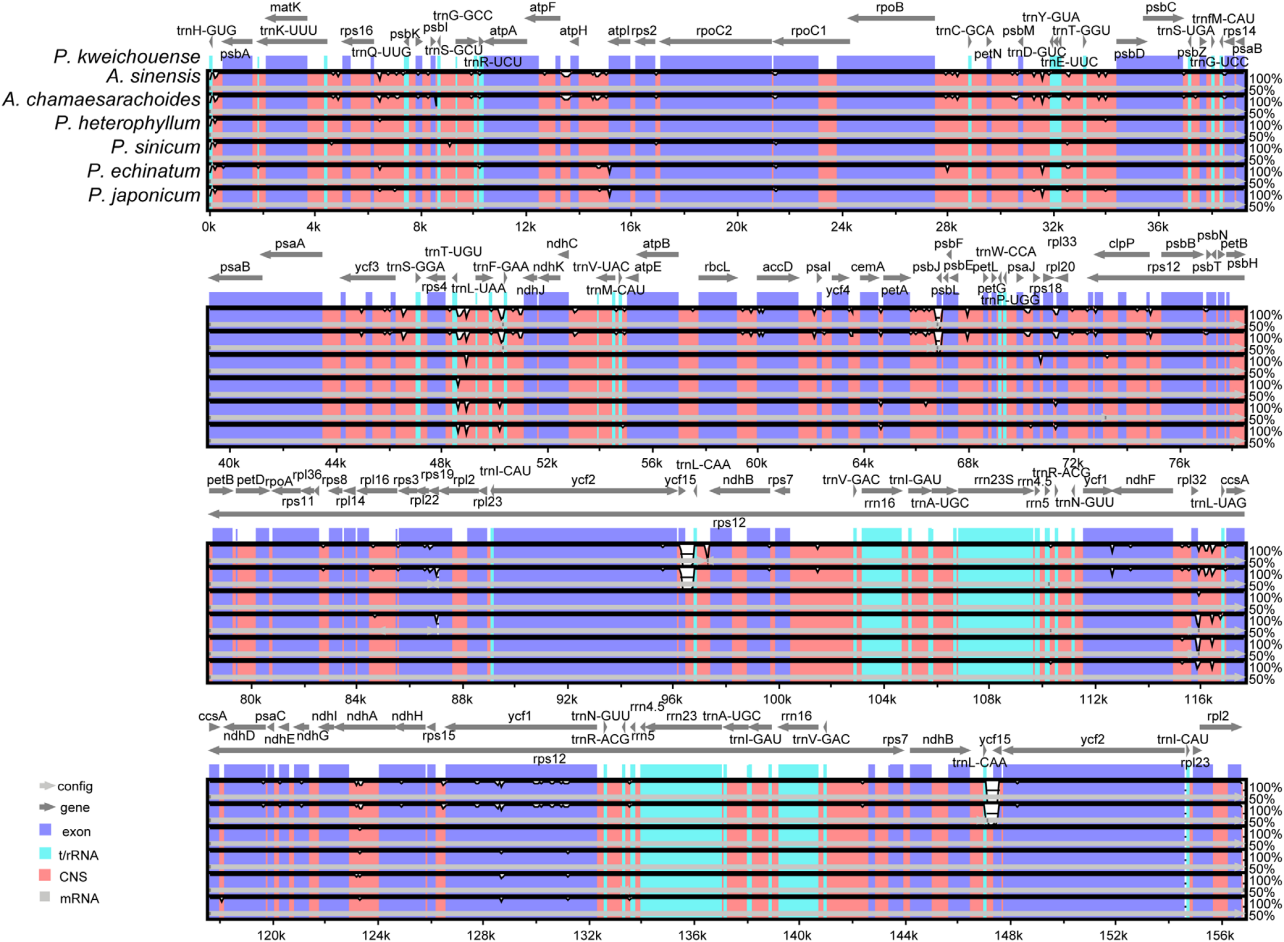
Visualization of the genome alignment of the chloroplast genomes of *Archiphysalis* and *Physaliastrum*, generated by mVISTA, using *P. kweichouense* as a reference. The *X*‐axis represents the coordinates of the chloroplast genome, whereas the *Y*‐axis represents the different species. Sequence similarity of aligned regions is displayed as horizontal bars, with similarity percentage ranging from 50% to 100%.

The annotated complete chloroplast genome of *Physaliastrum kweichouense* served as the reference sequence for global mVISTA visualization and comparative analysis. The results indicated that there was no gene rearrangement observed in both *Archiphysalis* and *Physaliastrum*. The coding regions (exons) across the seven species showed higher conservation compared to the noncoding regions (CNS), with the IR regions being more conserved than the LSC and SSC regions (Figure 6).

**FIGURE 7:**
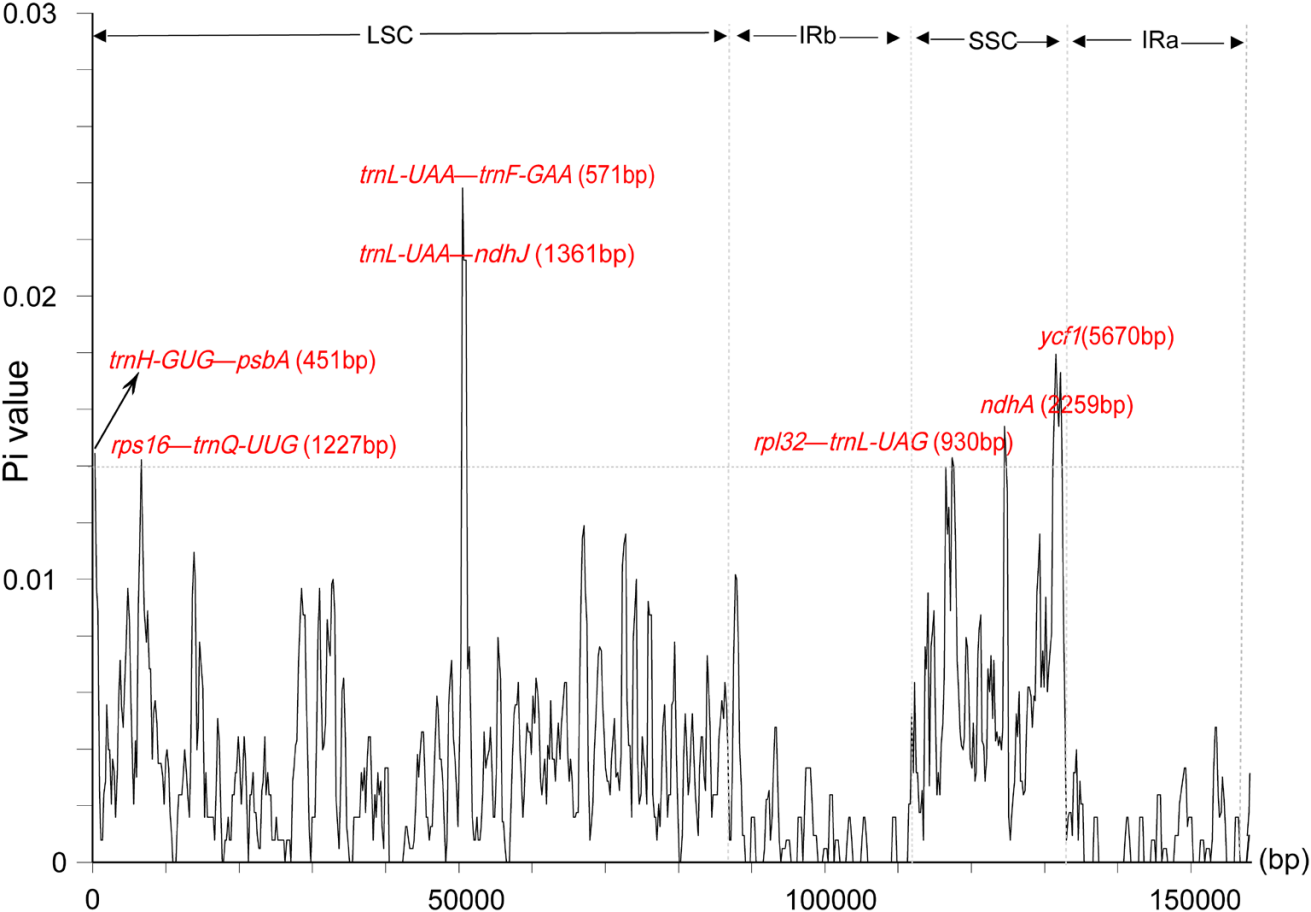
Nucleotide diversity (Pi) values calculated within a 600 bp sliding window across the whole chloroplast genomes of *Archiphysalis* and *Physaliastrum* samples. Genes exhibiting high Pi values (greater than 0.014) are annotated.

DnaSP identified a total of 774 polymorphic sites in the chloroplast genomes, with an average nucleotide diversity (Pi) value of 0.00330. Notably, the regions *trnH‐GUG*–*psbA*, *rps16*–*trnQ‐UUG*, *trnL‐UAA*–*trnF‐GAA*, *trnF‐GAA*–*ndhJ*, and *rpl32*–*trnL‐UAG*, along with the genes *ndhA* and *ycf1*, displayed significant variability, with Pi values exceeding 0.01400 (Figure 7, Table S1).

#### 3.4.3 Repeat Analysis

**FIGURE 8:**
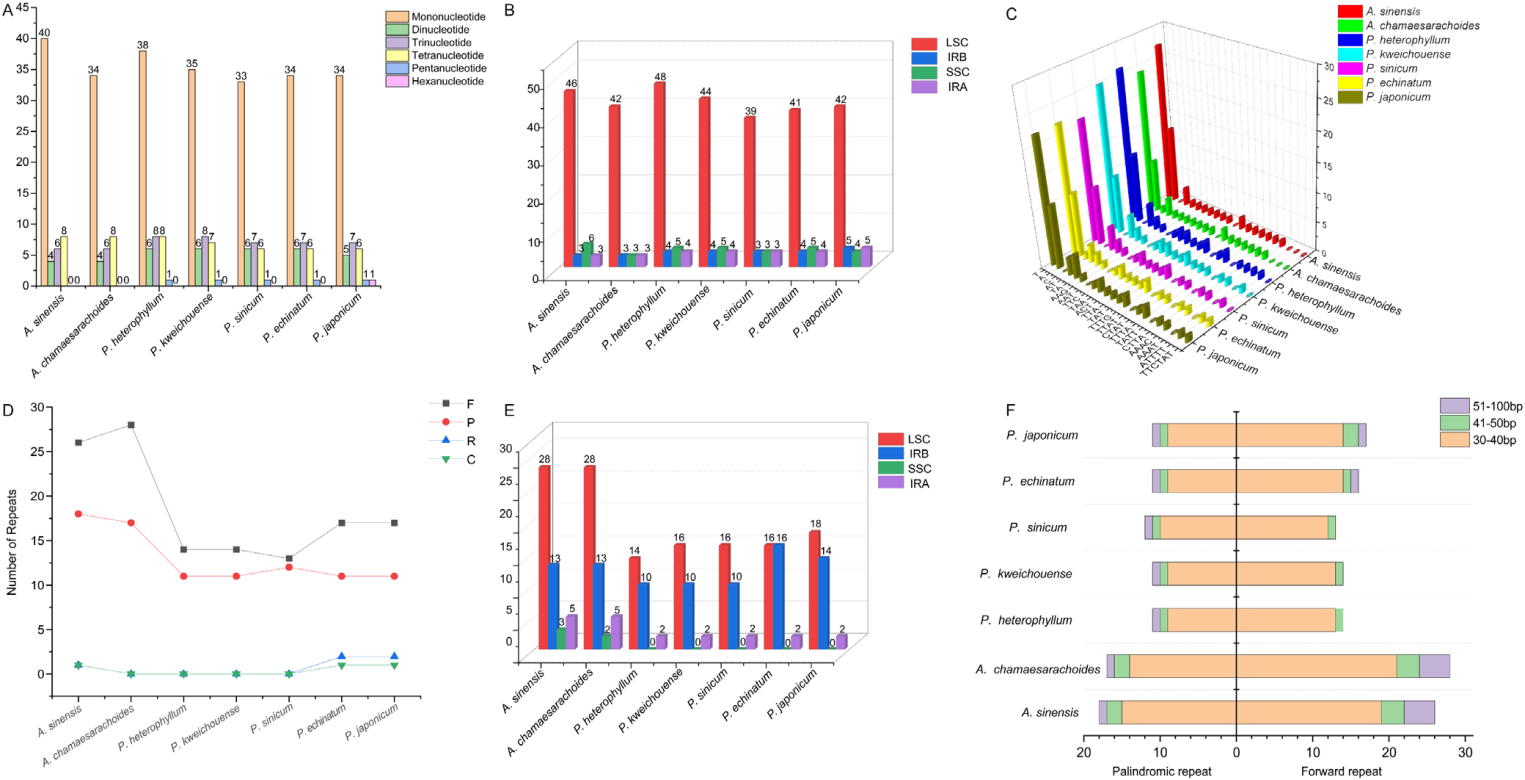
Analysis of repeat sequences in the cp genomes of *Archiphysalis* and *Physaliastrum*. (A) Types of SSRs. (B) Number of SSRs in the four genomic regions; (C) Distribution of SSR motifs across species. (D) Statistics of long repeat types: “C” for complementary, “F” for forward, “P” for palindromic, and “R” for reverse repeats. (E) Number of long repeats in the four genomic regions. (F) Comparison of palindromic and forward repeats across species.

A total of 52–61 SSR sites were detected in the complete chloroplast genomes of the studied species, with *Physaliastrum heterophyllum* showing the highest number (61) and *Archiphysalis chamaesarachoides* the lowest (52). Mononucleotide repeats were the most abundant (61.40%–68.97%), primarily composed of A/T repeats, while C repeats were only found in *A. chamaesarachoides* and *P. echinatum* . AT/TA repeats were the second most abundant (6.90%–12.96%). Pentanucleotide repeats were absent in *A. chamaesarachoides* and *A. sinensis* but present in all *Physaliastrum* species (1 each). Unique repeats, such as ATTTT and TTCTAT, were specific to certain species (Figure 8A–C). SSRs were predominantly located in the LSC region (73.58%–80.76%), with intergenic spacers (IGS) harboring the majority (56.60%–69.23%) (Table S2).

Long repeat sequences, including palindromic (P), forward (F), reverse (R), and complementary (C) repeats, were also identified. Overall, the two *Archiphysalis* species exhibit a significantly higher number of long repeats compared to the five *Physaliastrum* species, with counts ranging from 48 to 49 for *Archiphysalis* and 26–34 for *Physaliastrum*. Specifically, *A. sinensis* has the highest count at 49 long repeats, while *P. heterophyllum* has the lowest at just 26 (Table S3). Long repeat sequences were predominantly located in the LSC region (47.05%–58.33%) and IRb region (26.53%–47.06%). Palindromic and forward repeats with lengths of 30–40 bp were the most abundant. Notably, *A. chamaesarachoides*, *P. heterophyllum* , *P. kweichouense*, and *P. sinicum* lacked reverse and complementary repeats, whereas *A. sinensis* had one reverse repeat and one complementary repeat. Additionally, *P. echinatum* and *P. japonicum* each had two reverse repeats and one complementary repeat (Figure 8D–F).

#### 3.4.4 Codon Usage Bias Analysis

The total number of codons in the protein‐coding genes of the chloroplast genomes ranged from 20,949 to 20,960 across the seven species. Analysis of the 64 codons encoding 20 amino acids revealed that leucine (Leu), arginine (Arg), and serine (Ser) were the most abundant, each represented by six codons, whereas methionine (Met) and tryptophan (Trp) were the least abundant, with only one codon each. Leu was the most frequently used amino acid, accounting for 10.50%–10.54% of total codons across species. Ser ranked second (7.49%–7.52%), followed by Arg (5.98%–6.11%). The number of codons was relatively conserved, with no significant differences among species.

**FIGURE 9:**
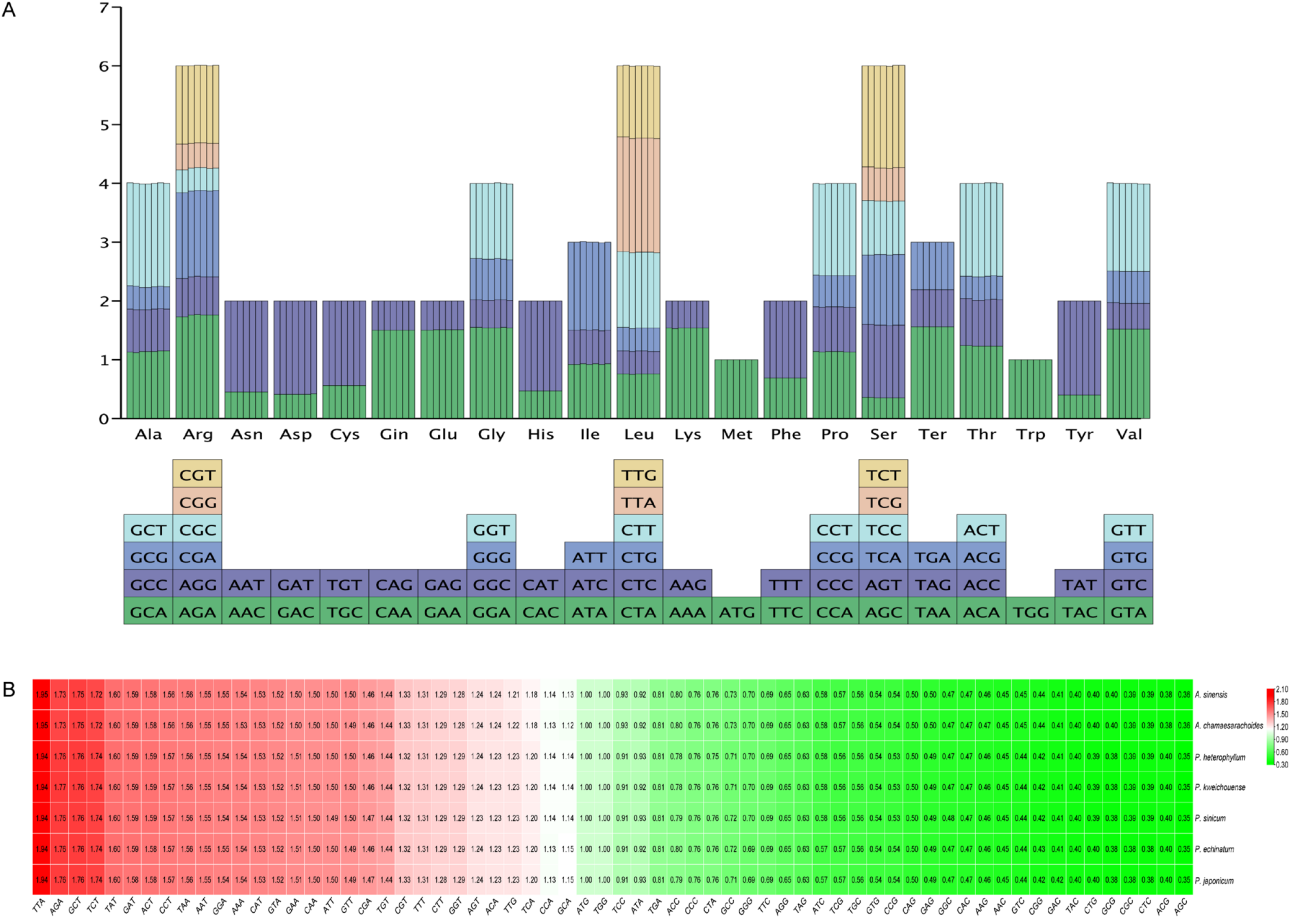
Analysis of codon usage bias in *Archiphysalis* and *Physaliastrum*. (A) Codon content for 20 amino acids and stop codons in the cp genomes. Each bar represents a species, from left to right: *A. sinensis* , *A. chamaesarachoides*, *P. heterophyllum* , *P. kweichouense*, *P. sinicum*, *P. echinatum* , *P. japonicum* . (B) Relative synonymous codon usage (RSCU) values across the cp genomes, with a color gradient from blue (low) to red (high).

Codon usage bias was assessed using the RSCU values. Except for Met and Trp, which had RSCU values of 1 (indicating no bias), the remaining amino acids showed varied RSCU values: 30 codons had RSCU values greater than 1, and 32 had values less than 1 (Figure 9; Table S4).

The complete chloroplast genomes of seven species were analyzed using the rscu.py script, identifying 52 protein‐coding genes in each genome. The length of the protein‐coding regions ranged from 62,847 to 62,880 bp, with GC content varying between 38.03% and 38.14%. The GC content was highest in the first codon position (46.22%–46.34%), followed by the second (38.17%–38.27%) and third positions (29.69%–29.81%).

**FIGURE 10:**
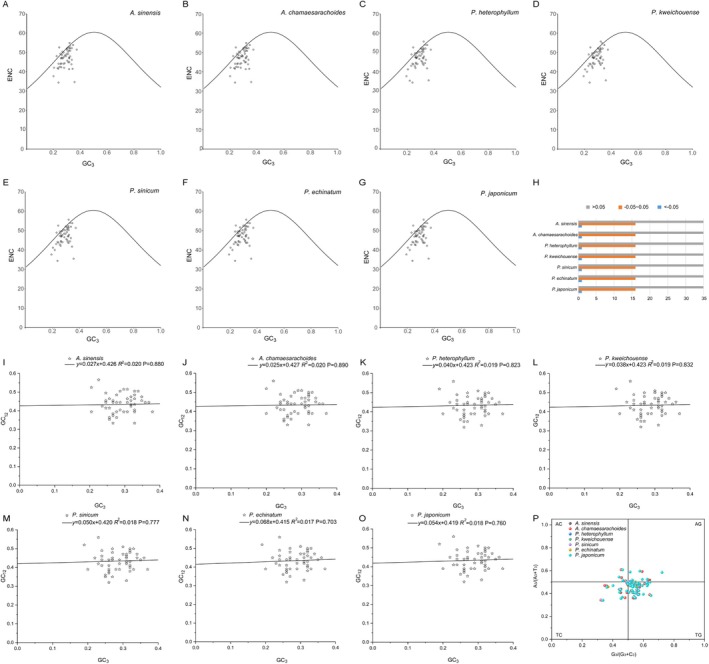
Integrated analysis of ENC, Neutrality, and PR2 bias in *Archiphysalis* and *Physaliastrum*. (A–G) ENC‐GC_3s_ plots. (H) Number of genes across different ENC ratio frequencies. (I–O) Neutrality plots. (P) PR2‐bias plot.

The ENC‐GC_3s_ plots indicated that most genes did not fall along the expected curve, suggesting a significant discrepancy between the actual ENC values and the expected ENC values (Figure 10A–G). Further analysis using the ENC ratio frequency revealed that among the 52 analyzed genes, all seven species exhibited 15 genes with ENC ratios within the range of −0.05 to 0.05, indicating that these genes are relatively close to the expected ENC values and are significantly influenced by mutation. In contrast, the majority of the remaining 37 genes fall outside this range, indicating a stronger influence of natural selection on their codon usage (Figure 10H; Table S5). Overall, it can be concluded that, for the genera *Physaliastrum* and *Archiphysalis*, the codon usage preference of the vast majority of genes is primarily shaped by selection, while a smaller subset of genes is more impacted by mutation.

Neutrality plots demonstrated that the correlation between GC12 and GC3 content was not significant (*p* = 0.703–0.890), indicating distinct base compositions in the first and second codon positions compared to the third position. This further supports the notion that codon usage is predominantly influenced by selection (Figure 10I–O; Table S6). Additionally, the PR2‐bias plot revealed an uneven distribution of the 52 genes across four regions, with most genes concentrated in the lower right quadrant (Figure 10P; Table S7). This pattern highlights a higher frequency of T over A and G over C usage in the third codon position.

### 3.5 Selection Pressure Analysis of Protein Coding Genes

Seventy‐nine unique protein‐coding genes (PCGs) without duplication were extracted, and their Ka/Ks values were analyzed to identify significant selection pressures. During this process, we excluded genes with Ka/Ks values of “NA” (indicating unavailable data) or 0, as these values typically represent cases with no substitutions or complete matches and thus do not provide meaningful information about selection pressures. This filtering step ensured that only genes with measurable evolutionary dynamics were included in the final analysis.

**FIGURE 11:**
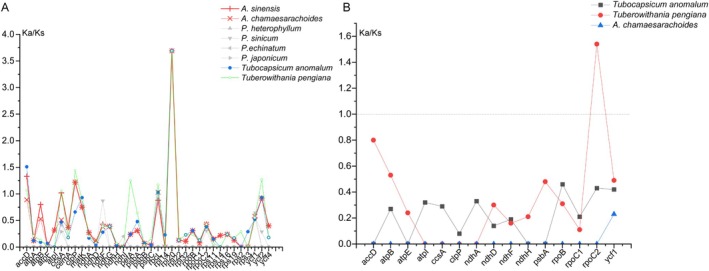
Ka/Ks Analysis of Protein‐Coding Genes. (A) Ratio of 36 genes with valid Ka/Ks values across four genera, using *Physaliastrum kweichouense* as the reference. (B) Ratio of 15 genes with valid Ka/Ks values in three Withaninae genera, using *Archiphysalis sinensis* as the reference.

Using *Physaliastrum kweichouense* as the reference, we identified 36 protein‐coding genes with valid Ka/Ks values. Among these, *Tubocapsicum anomalum* had three genes with Ka/Ks > 1: *accD* (1.51), *rbcL* (1.03), and *rpl20* (3.69). *Archiphysalis sinensis* and *A. chamaesarachoides* had four and three such genes, respectively, with the former including *accD* (1.33), *ccsA* (1.02), *clpP* (1.22), and *rpl20* (3.69), and the latter having *clpP* (1.22), *rbcL* (1.03), and *rpl20* (3.69). *Tuberowithania pengiana* had seven genes with Ka/Ks > 1: *accD* (1.07), *ccsA* (1.06), *clpP* (1.44), *petA* (1.25), *rbcL* (1.74), *rpl20* (3.69), and *ycf2* (1.27). These results suggest that these genes may be under positive selection, where nonsynonymous mutation rates exceed synonymous mutation rates. For the remaining protein‐coding genes in all species, Ka/Ks values were less than 1, indicating purifying selection, where nonsynonymous mutations are eliminated to maintain functional conservation (Figure 11A; Table S8).

When using *Archiphysalis sinensis* as the reference protein sequence, we identified 15 protein‐coding genes with valid Ka/Ks values. Among these, only the *rpoC2* gene in *Tuberowithania pengiana* exhibited a Ka/Ks value greater than 1, while the Ka/Ks values of the other genes were all less than 1, suggesting purifying selection (Figure 11B; Table S8). This further supports the prevalence of purifying selection in maintaining gene function across species.

Combining the Ka/Ks analysis with the phylogenetic tree in Figure 3, we observed that species within the same branch exhibited higher similarity in their protein‐coding sequences. This indicates that the closer the evolutionary relationship between species, the higher the conservation of their gene sequences. The consistency between the Ka/Ks analysis results and the phylogenetic relationships further supports the reliability of Ka/Ks values as an effective tool for assessing selection pressures in gene evolution.

## 4 Discussion

This study presents the complete chloroplast genomes of nine Chinese taxa, which include two species of *Archiphysalis*, five species of *Physaliastrum*, as well as *Tuberowithania pengiana* and *Tubocapsicum anomalum*. To elucidate the phylogenetic position of *A. sinensis* , we downloaded 47 chloroplast genomes from the NCBI database. Our genomic phylogenetic analysis effectively resolves the relationships between the genera *Archiphysalis* and *Physaliastrum*, supporting their distinct generic status. Notably, our findings indicate that *A. sinensis* clusters with *A. chamaesarachoides* within the subtribe Withaninae, which is consistent with their morphological traits. This close relationship is further corroborated by analyses of chloroplast gene characteristics. A detailed comparative analysis of the chloroplast genomes revealed significant differences in gene features between *Archiphysalis* and *Physaliastrum*, particularly regarding gene count, gene structure, and the lengths of gene duplications. The intergeneric differences in chloroplast gene features were markedly greater than the intrageneric differences. Our study provides genomic‐level support for the classification of species within these two genera and further highlights the close relationship between *A. chamaesarachoides* and *A. sinensis* .

### 4.1 Phylogenomic Comparison of Chloroplasts in *Physaliastrum* and *Archiphysalis*

This study successfully assembled the complete chloroplast genomes of two species of *Archiphysalis* and five species of *Physaliastrum*, along with two related genera, *Tuberowithania* (*T. pengiana*) and *Tubocapsicum* ( *T. anomalum* ), using second‐generation sequencing technology. With the exception of *P. yunnanense* , which exhibited poor quality and could not be well assembled, all species of *Archiphysalis*, *Physaliastrum*, *Tubocapsicum*, and *Tuberowithania* distributed in China were included in our research.

Through a detailed analysis of the chloroplast genomes of the two *Archiphysalis* species and five *Physaliastrum* species, we found that intergeneric differences in chloroplast gene features were significantly greater than the intrageneric differences. Divergence time analysis indicates that *Physaliastrum* split from *Archiphysalis* during the middle Eocene, approximately 41.2 million years ago (95% HPD: 38.6–43.5 Mya; Figure 4). As a result, there are notable differences in chloroplast gene features between *Archiphysalis* and *Physaliastrum*. For instance, our research revealed that the chloroplast genomes of *Archiphysalis* contain 85 protein‐coding genes, while those of *Physaliastrum* have 87. This difference arises from the absence of two copies of the *ycf15* gene in the *Archiphysalis* species (Table 2).

In terms of genomic structure, we also identified distinctions between the two genera. Overall, the Large Single Copy (LSC) region of *Archiphysalis* species is about 450 bp longer than that of *Physaliastrum*, while the Inverted Repeat (IR) region is approximately 850 bp shorter (Table 1). Analysis of repetitive sequences indicated that the primary differences between the two genera lie in the Forward repeats and Palindromic repeats, with the most significant variations occurring in lengths of 30–40 bp. These two types of repeats are predominantly located within the LSC region of *Archiphysalis*, which may explain why this region is considerably longer than that of *Physaliastrum* (Figure 8D–F). The shorter IR region in *Archiphysalis* can also be attributed to the absence of the two *ycf15* genes, while in *Physaliastrum*, the presence of these genes and their adjacent intergenic regions contributes approximately 900 bp to their IR length.

Results from the IR contraction and expansion analysis show that the boundary genes are identical in both genera: *rps19* spans the LSC/IRb boundary, *ψycf1* spans the IRb/SSC boundary, and *ycf1* spans the SSC/IRa boundary. The IRa/LSC boundary is located between the *rpl2* and the *trnH‐GUG* genes, indicating a high degree of conservation. In species of *Physaliastrum*, the *ycf1* gene and *ψycf1* pseudogene in the IR region extend into the SSC region by 19–23 bp, while in the genus *Archiphysalis*, both extend into the SSC region by 29 bp (Figure 5).

The mVISTA analysis reveals genetic variations between the species of *Physaliastrum* and *Archiphysalis*, with the most significant difference being the absence of two *ycf15* genes in the latter. In terms of gene type, the variation points are primarily located in the exons, followed by noncoding regions (CNS). Regarding gene distribution, the IR region exhibits very few variations aside from the absence of *ycf15*, making it the most conserved region (Figure 6). The DnaSP analysis identified four high mutation regions in the LSC and three in the SSC, each with Pi values greater than 0.014. Based on the lengths of these segments, these regions can serve as potential barcoding segments for the two genera (Figure 7).

The analysis of codon usage preference shows that both intra‐ and inter‐generic codon usage among the species of these two genera is largely consistent, with no significant differences observed (Figure 9). ENC‐GC3s plots and Neutrality plots indicate that most genes in these species are influenced more strongly by natural selection regarding their codon usage, while a smaller subset is more affected by mutation. The PR2‐bias plot highlights a higher frequency of thymine (T) over adenine (A) and guanine (G) over cytosine (C) usage in the third codon position (Figure 10).

Moreover, in the analysis of selection pressure, we found that protein‐coding sequences of species on the same branch were more similar, indicating that these species have close phylogenetic relationships and may have experienced similar selection pressures during evolution. Overall, among the 37 protein‐coding genes with valid Ka/Ks values, only eight genes exhibit Ka/Ks values greater than 1, indicating they are subject to positive selection, whereas 29 genes exhibit Ka/Ks values less than 1, reflecting purifying selection that eliminates nonsynonymous mutations to maintain functional conservation. These findings further support the prevalence of purifying selection in preserving gene function across species, consistent with many previous studies (Bai et al. 2022; Meng et al. 2023; Palmé et al. 2008; Zhang et al. 2023).

Based on the above analysis, the chloroplast genomes of species within the genera *Physaliastrum* and *Archiphysalis* display relatively minor differences and are largely consistent. However, notable distinctions exist between the two genera concerning gene count, gene structure, and the lengths of gene duplications. These findings provide genomic‐level support for the classification of species within these genera and further highlight the close relationship between *A. chamaesarachoides* and *A. sinensis* .

### 4.2 Phylogenetic Analysis and Taxonomic Implications

Our research indicates that chloroplast genome data effectively resolve the relationships between the genera *Archiphysalis* and *Physaliastrum* (Figure 3). The phylogenetic tree based on this data shows that all species within *Physaliastrum* cluster into a single evolutionary branch, receiving high support (BS = 100, PP = 1). Similarly, the two species of *Archiphysalis* also cluster together (BS = 100, PP = 1) and form an independent evolutionary branch within the subtribe Withaninae, alongside the genera *Tuberowithania* and *Tubocapsicum* (BS = 93, PP = 1).

The initial molecular phylogenetic study by Li et al. (2013) indicated a distant relationship between *Physaliastrum heterophyllum* and *Archiphysalis chamaesarachoides*, with the former in the subtribe Physalinae and the latter in the subtribe Withaninae. This relationship has been supported by subsequent studies (Deanna et al. 2019; Huang et al. 2023; Wang et al. 2024). Deanna et al. (2019) utilized four DNA markers (ITS, *LEAFY*, *trnL‐F*, and *waxy*) to analyze the tribe Physalideae, including both species of *Archiphysalis* (*A. chamaesarachoides* and *A. sinensis* ) and three species of *Physaliastrum* ( *P. heterophyllum* , *P. japonicum* , and *P. echinatum* ). The phylogenetic tree revealed that the two *Archiphysalis* species did not cluster together; instead, *A. chamaesarachoides* grouped with *Tubocapsicum anomalum*, falling within one of its two major branches of the subtribe Withaninae, while the type species *A. sinensis* clustered with the three species of *Physaliastrum*, forming a basal branch of the subtribe Physalinae. Although this study highlighted distinct phylogenetic positions for the two *Archiphysalis* species, it still supports the generic status of both *Archiphysalis* and *Physaliastrum*. Notably, the unexpected result of *A. sinensis* not clustering with *A. chamaesarachoides* is significant, given their similar fruit morphology. Furthermore, it is important to note that in Deanna's study, the sequence for *A. sinensis* was only available for the *LEAFY* fragment, and phylogenetic positions varied when constructed independently from different molecular fragments. For example, samples of *Withania* clustered together in trees constructed from the ITS and *trnL‐F* fragments but did not cluster in trees based on the *waxy* and *LEAFY* fragments.

In 2023, Huang utilized nearly 1700 orthologous nuclear genes from transcriptomic/genomic datasets to reconstruct a highly resolved phylogenetic tree for the Solanaceae family. Their study included only *Archiphysalis chamaesarachoides* and *Physaliastrum heterophyllum*, confirming their phylogenetic positions as consistent with Li et al. (2013), thereby supporting their independent status within the subtribes Physalinae and Withaninae, respectively.

In 2024, Wang et al. (2024) published a new genus, *Tuberowithania*. While using fragments from Deanna's work, they revealed a different systematic position for *Archiphysalis*. Their results indicated that the inclusion of *Tuberowithania* shifted the systematic placement of *A. chamaesarachoides* within the subtribe Withaninae. Additionally, *A. sinensis* , previously grouped with the *Physaliastrum* species in the subtribe Physalinae, has now been reclassified to cluster with the genera *Tubocapsicum*, *Discopodium*, *Nothocestrum*, and three species of *Withania*, forming an evolutionary branch within Withaninae. Notably, *A. sinensis* is still represented only by the *LEAFY* gene, and in trees based on this gene, it clusters with species of *Physalis* L. It is important to highlight that constructing matrices based on *LEAFY* fragments is challenging due to difficulties in obtaining a reliable aligned matrix. The authors also noted that *A. chamaesarachoides* exhibits significantly more shared base variations with *Tuberowithania* than with *Tubocapsicum*. Morphologically, *A. chamaesarachoides* appears more closely related to *Tuberowithani*a than to *Tubocapsicum*, as both possess 10 longitudinally thickened ribs on their fruiting calyx, further supporting its distinct taxonomic placement. Importantly, the authors cautioned that the relationships revealed in the deeper phylogenetic analysis may change with the inclusion of different molecular markers, consistent with Deanna et al. (2019). Therefore, we should remain cautious, as the phylogenetic positions of *A. chamaesarachoides* may vary with the addition of new samples or the use of different molecular markers.

This study, based on chloroplast genome phylogenetics, classifies the tribe Physalideae into three major clades: Iochrominae (Clade A), Withaninae (Clade B), and Physalinae (Clade C). Notably, our findings reveal that *A. sinensis* , the type species of *Archiphysalis*, clusters with *A. chamaesarachoides* within the subtribe Withaninae (BS = 100, PP = 1), rather than grouping with *Physaliastrum*, which aligns with our expectations based on morphological characteristics. This grouping forms a cohesive Clade B1 alongside *Tuberowithania pengiana* and *Tubocapsicum anomalum* (BS = 93, PP = 1). Furthermore, the five species of *Withania* also cluster together (BS = 100, PP = 1), associating with representative species of *Discopodium* and *Nothocestrum* to form Clade B2.

To minimize the impact of uniparental inheritance of chloroplast genes on the phylogenetic relationships of the two *Archiphysalis* species, we utilized GeneMiner (Xie et al. 2024) to extract four fragments (ITS, *LEAFY*, *trnL‐F*, and *waxy*) from our next‐generation sequencing data. These fragments were incorporated into a previous research matrix (Wang et al. 2024), and we constructed trees for each fragment as well as a combined matrix to observe any changes in the phylogenetic relationships of the two *Archiphysalis* species. Due to the absence of data for *A. sinensis* in three out of four fragments, we only utilized sequences from three *Archiphysalis* samples obtained in this study, which had nearly complete data for tree construction. The results showed that, regardless of whether we analyzed the combined data or each fragment separately, *A. chamaesarachoides* and *A. sinensis* consistently clustered together (BS = 96–100, PP = 1) within the Withaninae subtribe (Figures S1–S10).

Based on these findings, the authors suggest that while sample availability limits this study, the absence of samples from *Aureliana* Sendtn. and *Deprea* Raf. in this chloroplast phylogenetic analysis does not preclude the possibility that the phylogenetic relationships within Withaninae may change with the addition of chloroplast genome data from these genera. However, it is likely that the systematic relationships between the two *Archiphysalis* species will remain stable, even with more samples. To validate this, the authors plan to expand the representation of species within the Withaninae subtribe and increase sequencing depth, enabling the use of abundant orthologous genes for a more detailed examination of the phylogenetic relationships between *Archiphysalis* species and their relatives. This expanded dataset will enhance our understanding of the evolutionary dynamics and systematic placements within the Withaninae subtribe.

## Author Contributions

**Jiaju Xu:** data curation (equal), formal analysis (equal), methodology (equal), validation (equal), writing – original draft (equal), writing – review and editing (equal). **Zehuan Wang:** conceptualization (equal), funding acquisition (equal), investigation (equal), supervision (equal), validation (equal), writing – original draft (equal), writing – review and editing (equal). **Qianqian Zhong:** data curation (equal), formal analysis (equal), methodology (equal), validation (equal). **Li Yan:** data curation (equal), validation (equal), visualization (lead). **Sirong Yi:** investigation (equal), resources (equal). **Qingwen Sun:** funding acquisition (equal), supervision (equal).

## Ethics Statement

Experimental research and field studies on plants, including the collection of plant material, comply with relevant institutional, national, and international guidelines and legislation.

## Conflicts of Interest

The authors declare no conflicts of interest.

## Supporting information


Figure S1.

Figure T1.

Table S1.

## Data Availability

The data in this study are deposited in the NCBI (https://www.ncbi.nlm.nih.gov/), with accession numbers PV472654–PV472662 for complete chloroplast.
