# Supplementary material for: Genomic Phylogenetic Analysis of Physaliastrum and Archiphysalis (Solanaceae): Insights From Chloroplast Genomes Indicate Distinct Evolutionary Relationships
Source: Ecol Evol. 2025 Jul 7;15(7):e71762. doi: 10.1002/ece3.71762 (PMC12234071; doi:10.1002/ece3.71762)
Supplement: Supplementary file 1 — Figure S1. [file ECE3-15-e71762-s001.pdf]

Note: We constructed phylogenetic trees from FIGUREs S1-S10 using the Bayesian Inference (BI) and Maximum Likelihood (ML) methods. To facilitate comparison with existing research results, we referenced the sequences of all species involved in the phylogenetic trees based on four gene fragments (nrITS, *trnL-F*, *waxy*, *LFY*) from the literature "*Tuberowithania pengiana* (Withaninae, Physalideae, Solanaceae), a new species and genus from southwest Yunnan, China" (Wang et al. 2024), and incorporated these four gene fragments extracted by GeneMiner from our second-generation sequencing data.

We performed both joint tree construction of the four fragments and individual tree construction for each fragment. During the tree construction process, we used ModelFinder in PhyloSuite v1.2.3 to determine the best substitution models for the two analytical methods. The Bayesian method selected the best model based on the Bayesian Information Criterion (BIC), while the Maximum Likelihood method selected the best model based on the Akaike Information Criterion (AIC). Subsequently, we used MrBayes and IQ-TREE in PhyloSuite to construct the phylogenetic trees and applied the aforementioned selected models. MrBayes ran for 2,000,000 generations, and IQ-TREE ran for 1,000 standard generations, with all other parameters kept at their default values. The results are shown as follows:

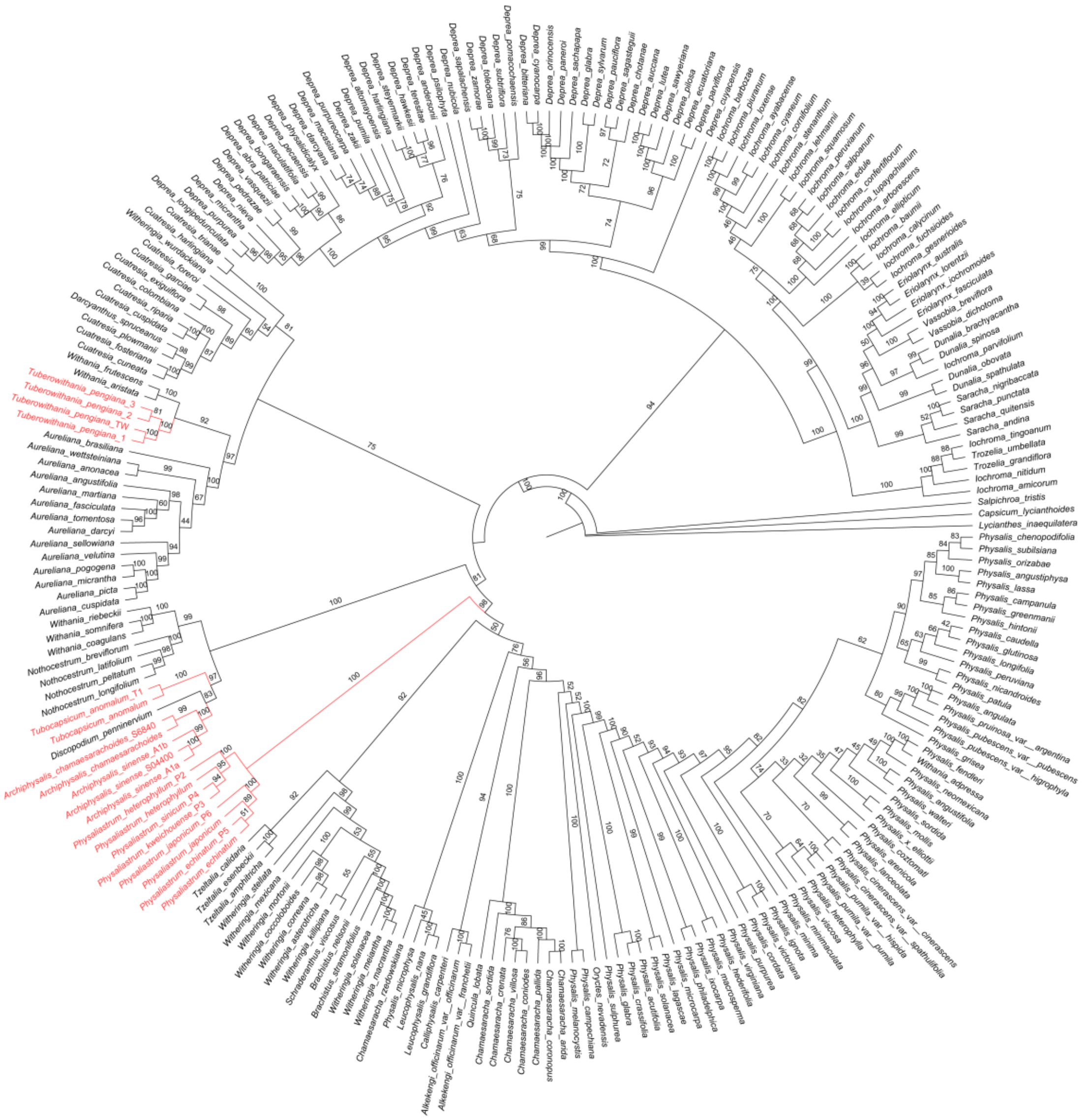

FIGURE S1. Phylogenetic relationships of the Physalideae tribe based on a Maximum Likelihood (ML) analysis of the combined dataset of four markers (ITS, *LFY*, *trnL-F*, and *waxy*).

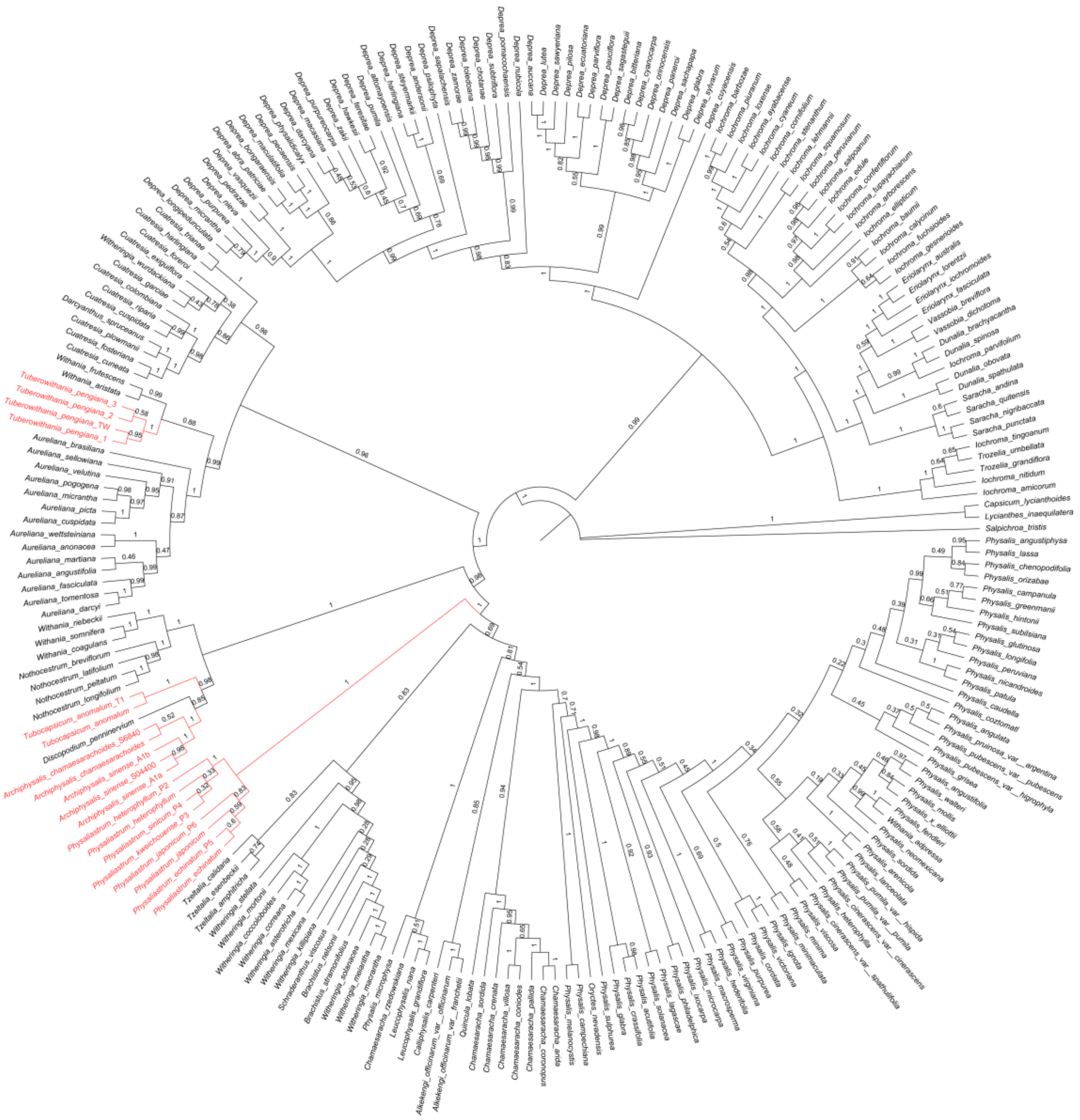

FIGURE S2. Phylogenetic relationships of the Physalideae tribe based on a Bayesian Inference (BI) analysis of the combined dataset of four markers (ITS, *LFY*, *trnL-F*, and *waxy*).

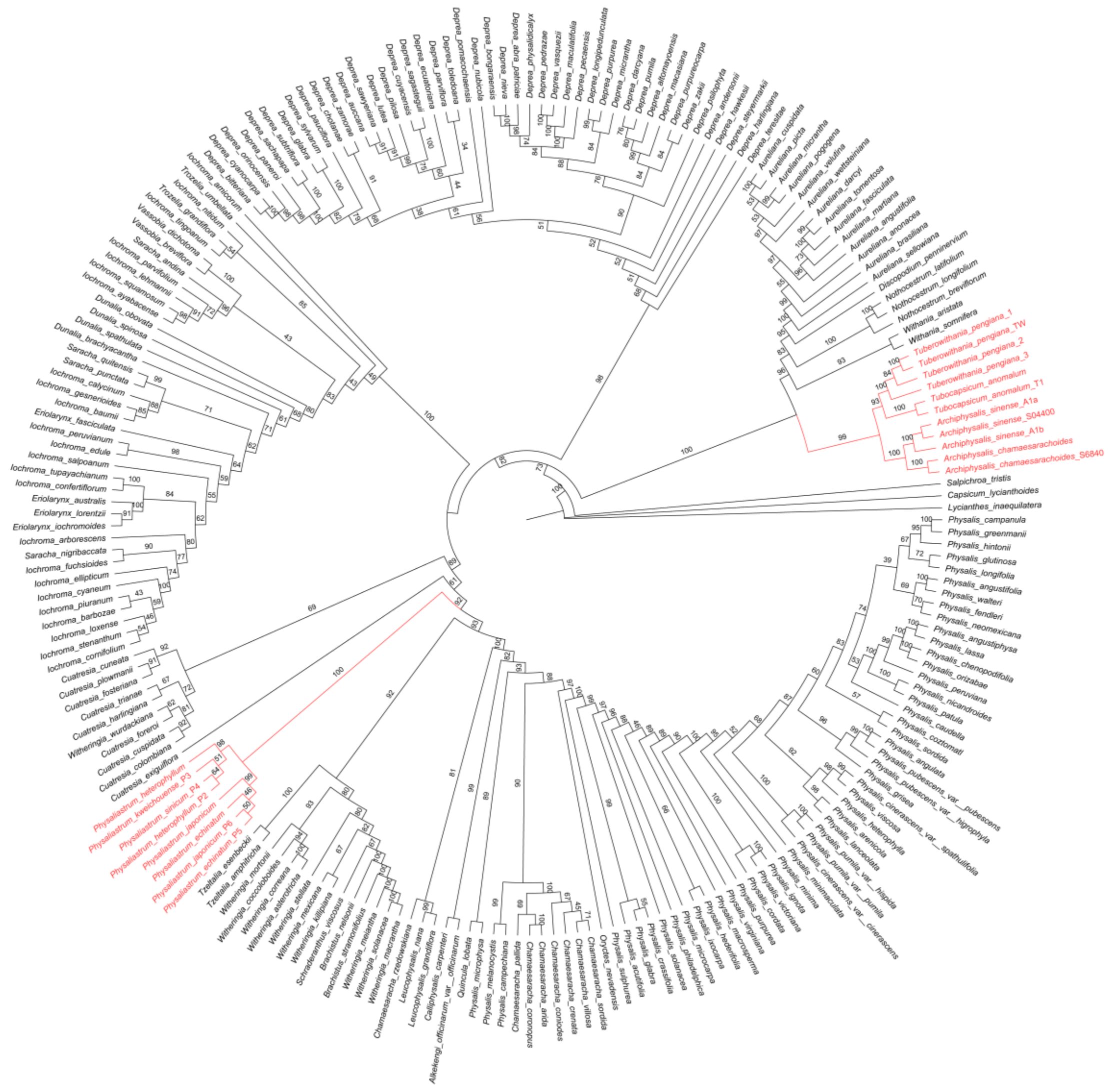

FIGURE S3. Phylogenetic relationships of the Physalideae tribe based on a Maximum Likelihood (ML) analysis of the ITS.

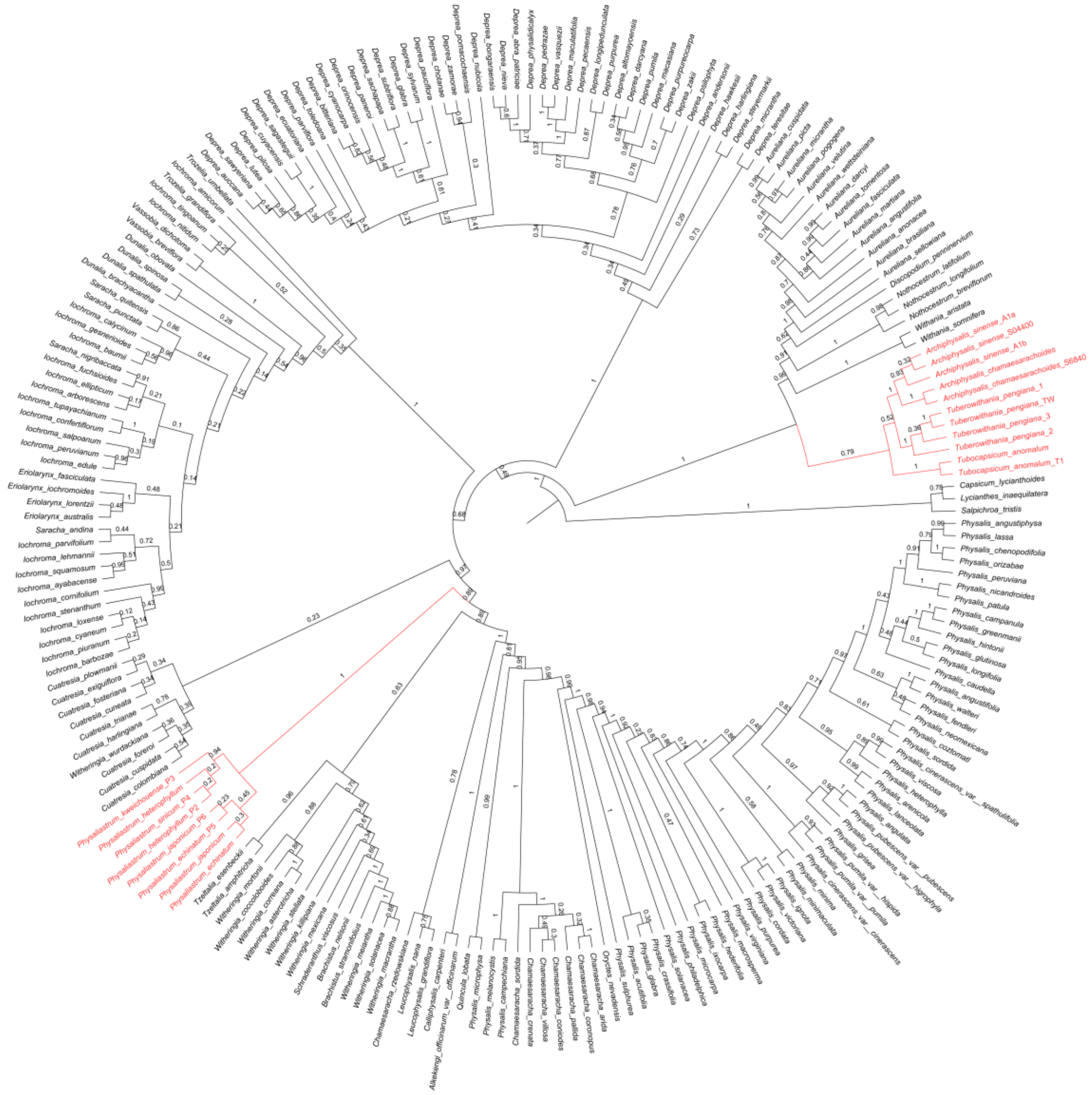

FIGURE S4. Phylogenetic relationships of the Physalideae tribe based on a Bayesian Inference (BI) analysis of ITS.

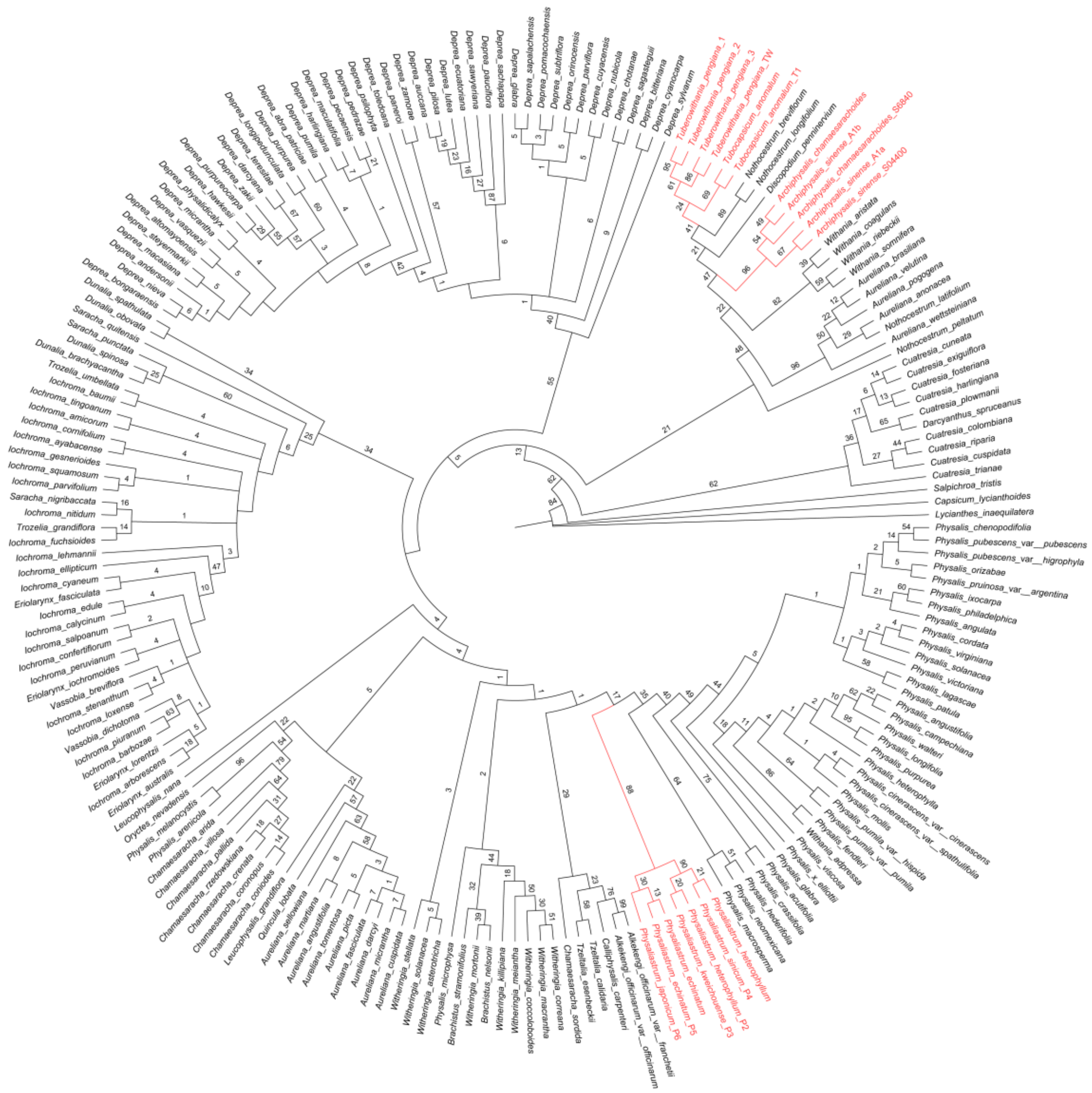

FIGURE S5. Phylogenetic relationships of the Physalideae tribe based on a Maximum Likelihood (ML) analysis of the *trnL-F*.

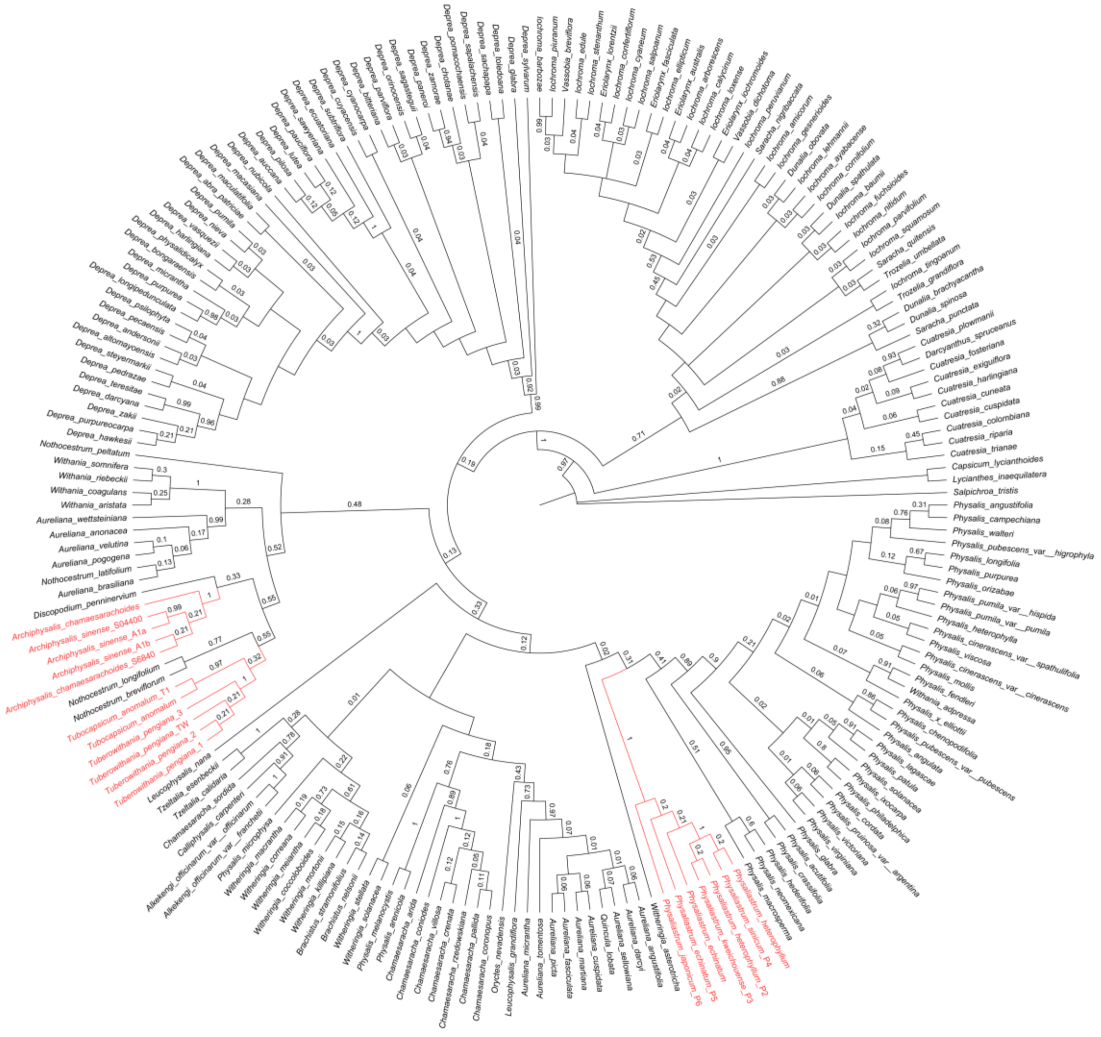

FIGURE S6. Phylogenetic relationships of the Physalideae tribe based on a Bayesian Inference (BI) analysis of *trnL-F*.

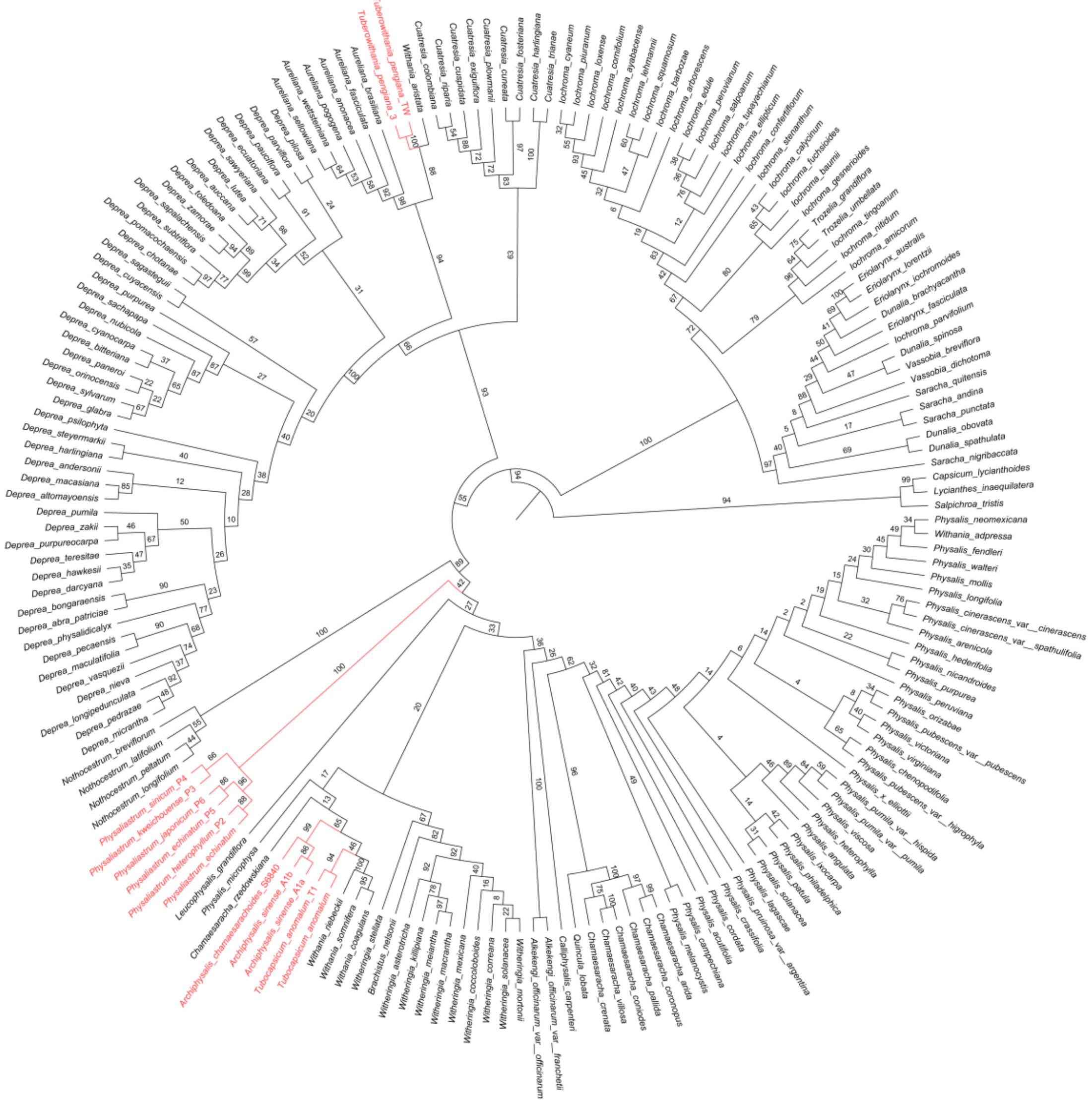

FIGURE S7. Phylogenetic relationships of the Physalideae tribe based on a Maximum Likelihood (ML) analysis of the *waxy*.

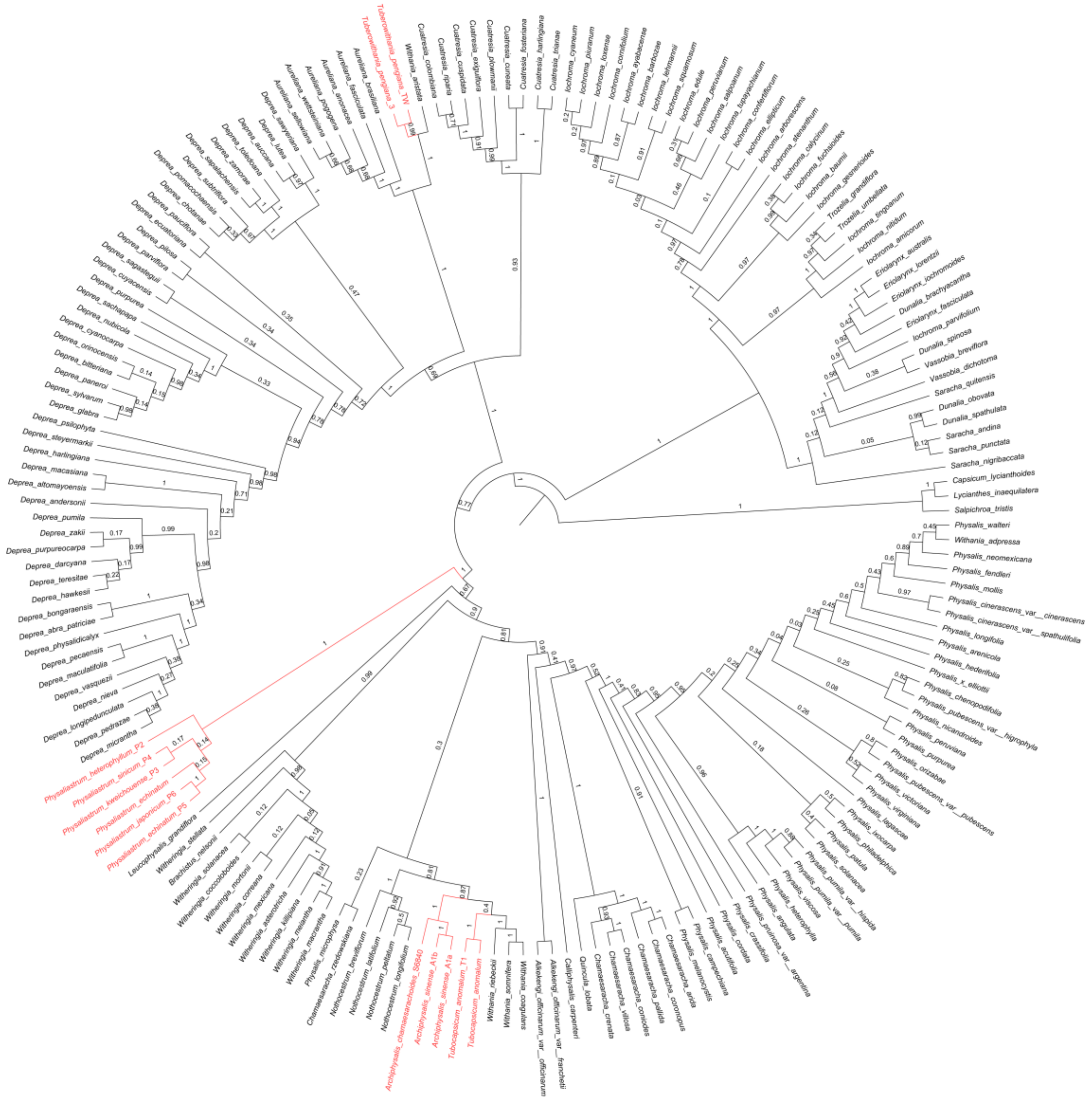

FIGURE S8. Phylogenetic relationships of the Physalideae tribe based on a Bayesian Inference (BI) analysis of *waxy*.

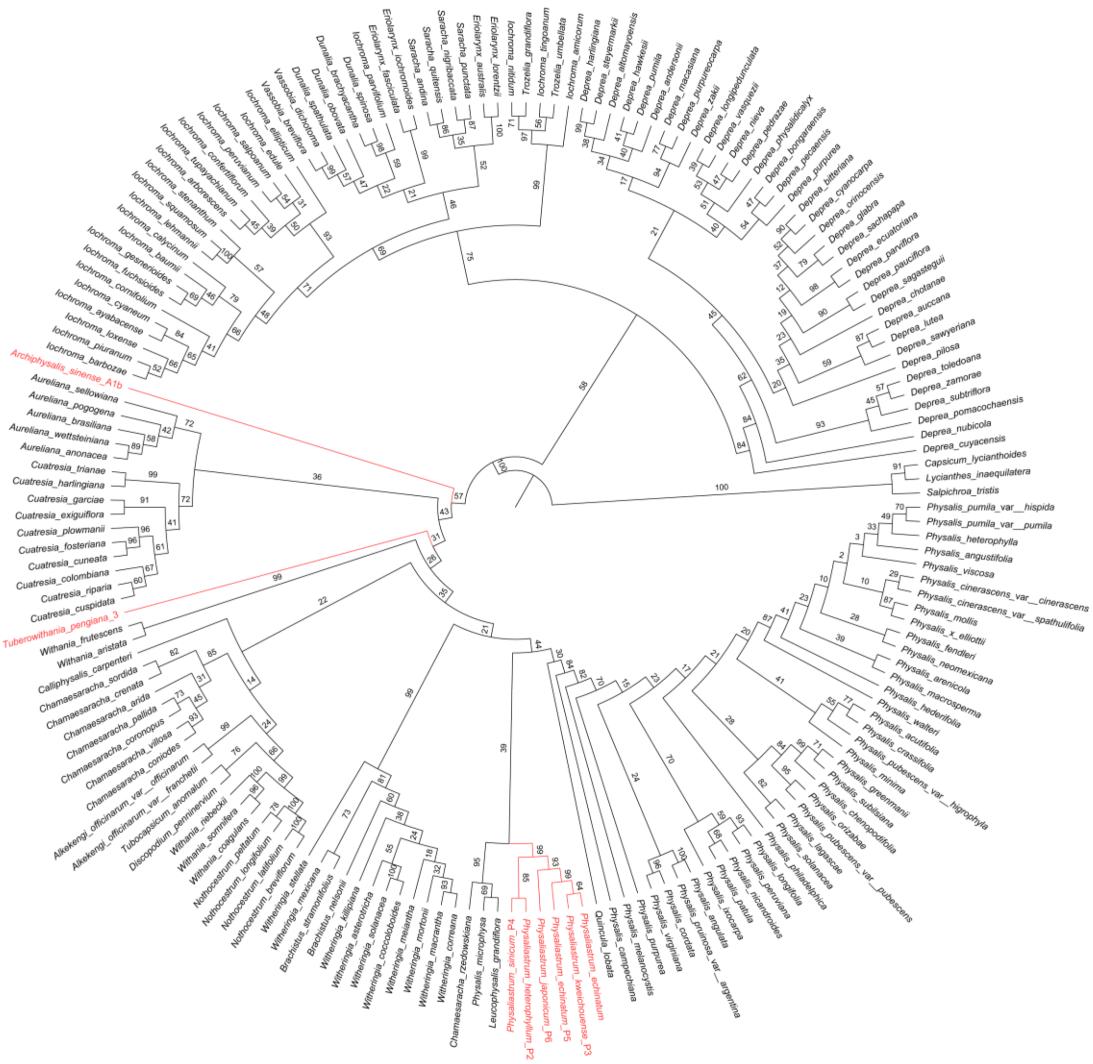

FIGURE S9. Phylogenetic relationships of the Physalideae tribe based on a Maximum Likelihood (ML) analysis of the *LFY*.

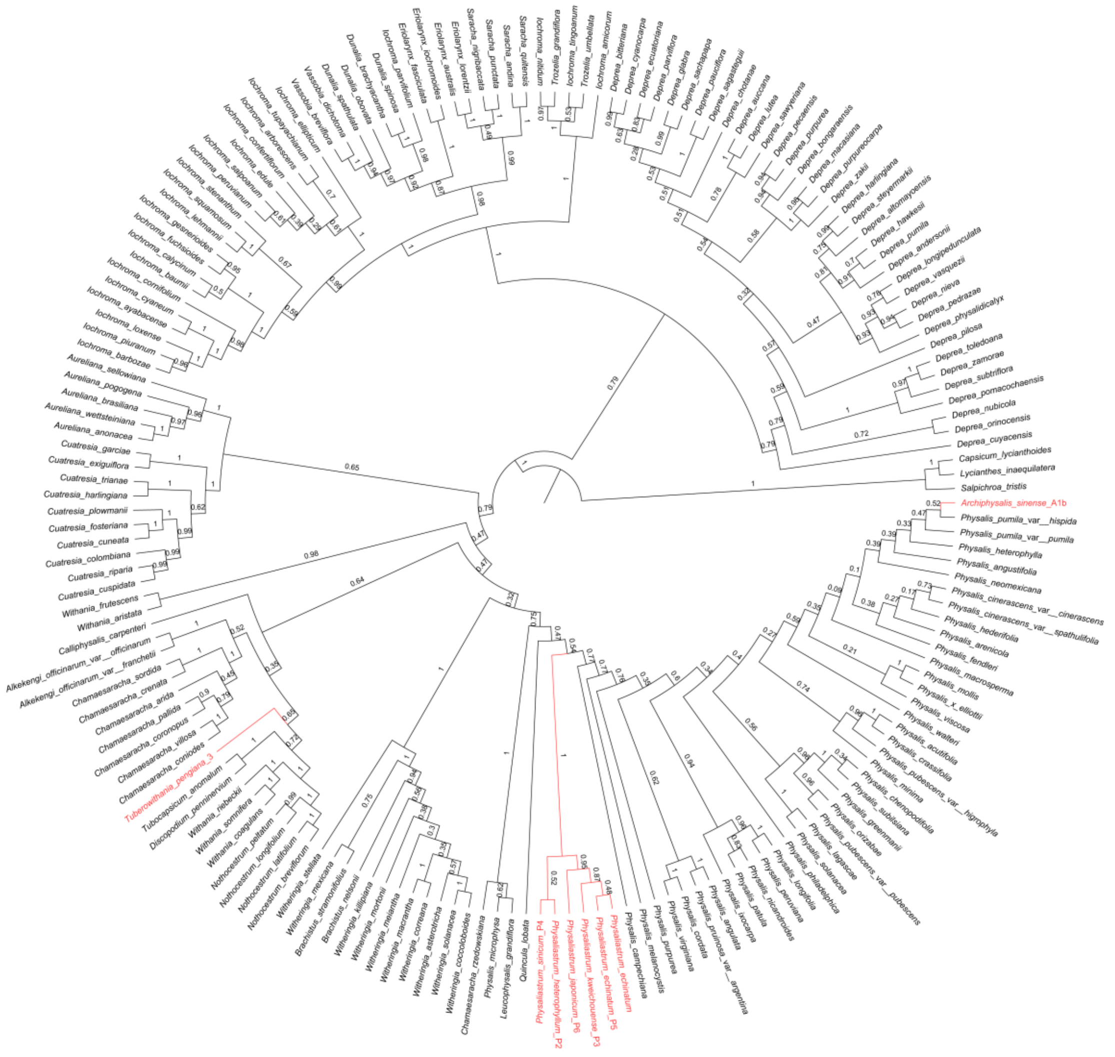

FIGURE S10. Phylogenetic relationships of the Physalideae tribe based on a Bayesian Inference (BI) analysis of *LFY*.
