# Supplementary material for: Genomic Phylogenetic Analysis of Physaliastrum and Archiphysalis (Solanaceae): Insights From Chloroplast Genomes Indicate Distinct Evolutionary Relationships
Source: Ecol Evol. 2025 Jul 7;15(7):e71762. doi: 10.1002/ece3.71762 (PMC12234071; doi:10.1002/ece3.71762)
Supplement: Supplementary file 2 — Figure T1. [file ECE3-15-e71762-s002.pdf]

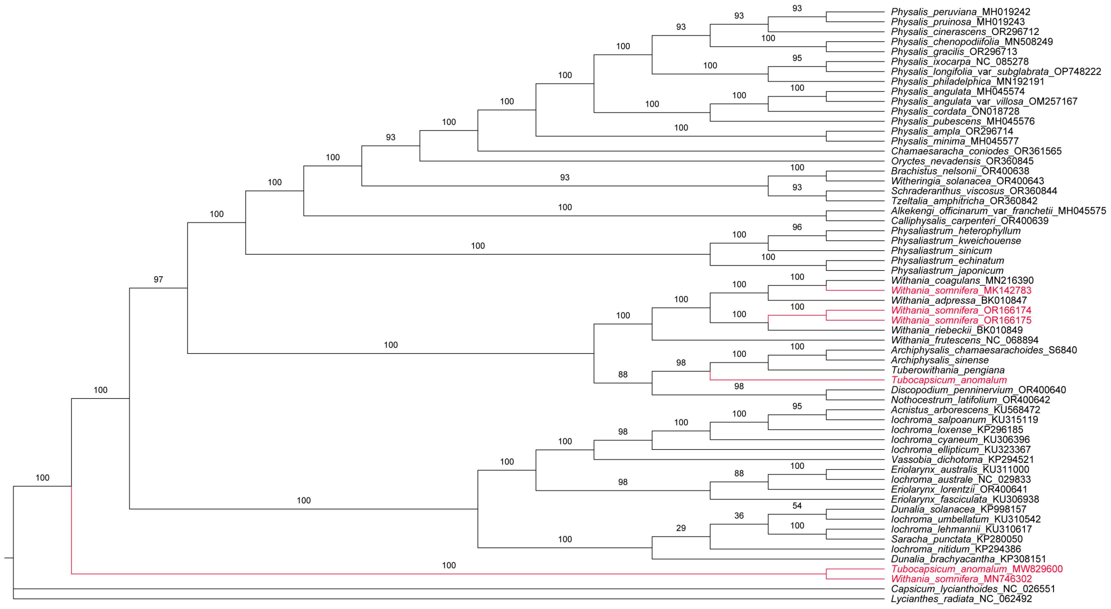

FIGURE T1. Phylogenetic relationships of the Physalideae tribe based on Maximum Likelihood (ML) analysis of the whole chloroplast genome of 60 taxa, including *Tuberowithania anomalum* (MW829600) and *Withania somnifera* (MN746302, MK142783, OR166175), in addition to the 56 taxa depicted in FIGURE 3. The sequences highlighted in red are all available sequences of *Tuberowithania* and *Withania* from the NCBI.

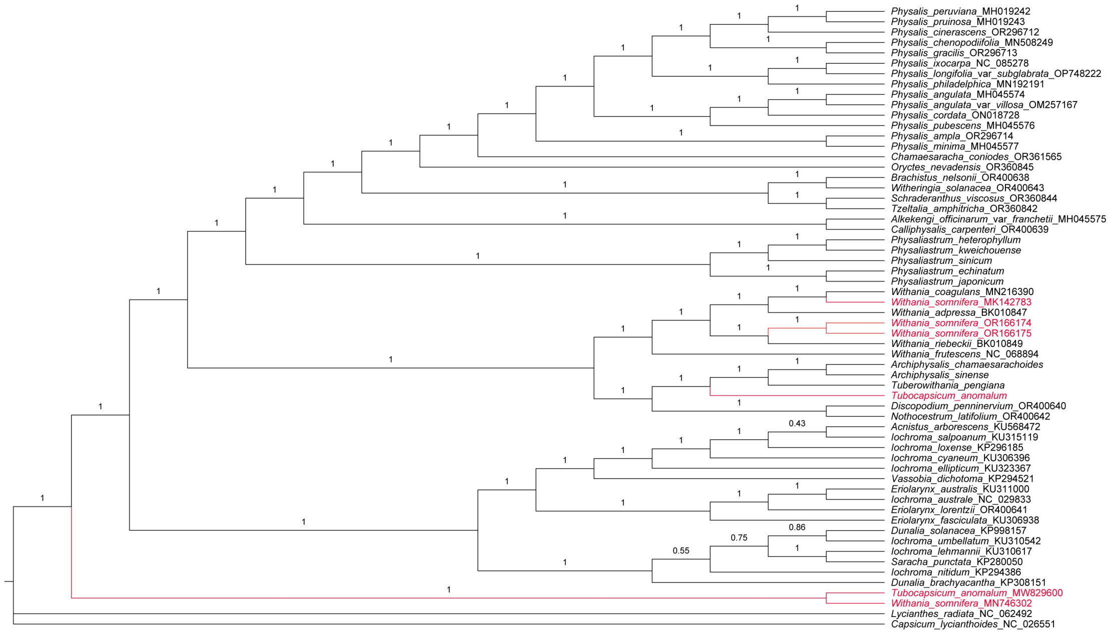

FIGURE T2. Phylogenetic relationships of the Physalideae tribe based on a Bayesian Inference (BI) analysis of the whole chloroplast genome of 60 taxa, including *Tuberowithania anomalum* (MW829600) and *Withania somnifera* (MN746302, MK142783, OR166175), in addition to the 56 taxa depicted in FIGURE 3. The sequences highlighted in red are all available sequences of *Tuberowithania* and *Withania* from the NCBI.
